# Immunomodulatory and anti-inflammatory potential of S-nitrosoglutathione (GSNO) in multiple sclerosis: a review of mechanisms and therapeutic perspectives

**DOI:** 10.3389/fimmu.2026.1797609

**Published:** 2026-08-19

**Authors:** Jeseong Won, Judong Kim, Avtar K. Singh, Inderjit Singh

**Affiliations:** 1 Department of Pathology and Laboratory Medicine, Medical University of South Carolina, Charleston, SC, United States; 2 Department of Pediatrics, Medical University of South Carolina, Charleston, SC, United States; 3 Pathology and Laboratory Medicine Service, Ralph H. Johnson Veterans Administration Medical Center, Charleston, SC, United States; 4 Research Service, Ralph H. Johnson Veterans Administration Medical Center, Charleston, SC, United States

**Keywords:** GSNOR inhibition, multiple sclerosis, neuroinflammation, oxidative stress, selective immunomodulation, S-nitrosoglutathione (GSNO), S-nitrosylation

## Abstract

Multiple sclerosis (MS) is a chronic autoimmune disorder of the central nervous system (CNS), characterized by sustained neuroinflammation, oxidative stress, demyelination, and progressive neurodegeneration. Although MS remains incurable, current disease-modifying therapies (DMTs) help reduce relapse frequency and delay disease progression. Recently developed injectable and infusion-based biologic DMTs offer targeted immunomodulation, but their clinical utility is limited by parenteral administration and the potential for immunogenicity. In contrast, small-molecule DMTs such as fingolimod and teriflunomide provide greater dosing convenience and flexibility; however, their broad immunosuppressive effects often lead to treatment-induced lymphopenia and increased susceptibility to infections. These limitations underscore the need for small-molecule DMTs that achieve selective immunomodulation without compromising systemic immune competence. S-nitrosoglutathione (GSNO), an endogenous nitric oxide (NO•) carrier molecule, has recently emerged as a promising candidate for immunomodulation. GSNO also mitigates oxidative stress, preserves blood-brain barrier integrity, and suppresses neuroinflammatory signaling via S-nitrosylation of key transcription factors such as Nrf2, HIF-1α, NF-κB, and STATs. The therapeutic potential of GSNO in MS is further supported by the development of reversible GSNO reductase (GSNOR) inhibitors, which elevate endogenous GSNO levels. These inhibitors have demonstrated favorable safety profiles in clinical trials for asthma and cystic fibrosis. In preclinical models of MS, GSNOR inhibitors suppress pathogenic Th1 and Th17 cells and IL-6-producing effector B cells while promoting regulatory T- and B-cell populations, thereby providing selective immunomodulation without broad immune suppression. As a result, they significantly reduce inflammation-driven demyelination. Building on this evidence, this review examines the GSNO/GSNOR axis as an emerging therapeutic target in MS, highlighting its mechanistic roles in selective immune modulation, the regulation of neuroinflammation, and the attenuation of oxidative stress. Furthermore, we discuss the translational potential of reversible GSNOR inhibitors, which have demonstrated favorable safety and tolerability in Phase I/II studies for asthma and cystic fibrosis, while emphasizing the need for future clinical evaluation of their therapeutic efficacy in MS.

## 1 Introduction

Multiple sclerosis (MS) is a chronic, immune-mediated disorder of the central nervous system (CNS) that affects over 2.8 million people worldwide. It is more prevalent in women and typically manifests between the ages of 20 and 40 (1). The etiology of MS is multifactorial, involving a complex interplay between genetic predisposition and environmental influences. Among the genetic risk factors, variations within the major histocompatibility complex (MHC) region, especially in human leukocyte antigen (HLA) genes, which play a key role in helping T cells recognize foreign antigens, have been strongly associated with increased susceptibility to MS (2, 3). Clinically, MS presents in different forms: most commonly as relapsing-remitting MS (RRMS), which may later evolve into secondary progressive MS (SPMS), or as primary progressive MS (PPMS) in a smaller proportion of patients (1, 4). Pathologically, MS is characterized by demyelinating lesions in the CNS driven by autoreactive T cells, B cells, and innate immune mechanisms, with oxidative stress and mitochondrial dysfunction contributing to neurodegeneration and disease progression (5).

MS remains an incurable disease, and because of its autoimmune-driven inflammatory nature, immune-targeted therapies have become the central focus of treatment. Acute relapses are typically managed with corticosteroids; however, their long-term use is constrained by severe, dose-dependent side effects. To overcome these limitations, more than 20 FDA-approved disease-modifying therapies (DMTs) are currently available, the majority of which act through immunosuppressive or immunomodulatory mechanisms. Among these, injectable (e.g., glatiramer acetate and interferon-β) and infusion-based biologic therapies (e.g., natalizumab and ocrelizumab) are generally considered more mechanism-based immunomodulators (**Table 1**). For instance, natalizumab prevents the infiltration of activated T cells into the CNS by targeting α4-integrin; glatiramer acetate (Copaxone), a synthetic peptide that mimics myelin basic protein (MBP), promotes anti-inflammatory immune responses by shifting T cell polarization from Th1 to Th2 phenotypes; and interferon-β regulates immune activity by downregulating proinflammatory cytokines such as IL-1 and TNF-α while enhancing anti-inflammatory mediators like IL-10 (6) (**Table 1**). Although these therapies represent significant progress in mechanism-based immunomodulation, their reliance on parenteral administration and potential to induce immunogenicity, through the development of neutralizing antibodies, limit their long-term efficacy and clinical utility.

**Table 1:** Comparison of current disease-modifying therapies and emerging gsnor inhibition for multiple sclerosis.

Therapeutic agents	Drug class	Route	Primary mechanism	Major advantages and limitations	References
Ocrelizumab (Ocrevus)	Anti-CD20 monoclonal antibody	IV	Depletion of CD20^+^ B cells	Advantages: High efficacy for RRMS and PPMS; infrequent dosing Limitations: Broad B-cell depletion, infection risk, hypogammaglobulinemia	(396–398)
Ofatumumab (Kesimpta)	Anti-CD20 monoclonal antibody	SC	Depletion of CD20^+^ B cells	Advantages: Self-administered; high efficacy Limitations: Continuous B-cell depletion; infection risk	(399, 400)
Dimethyl fumarate (Tecfidera)	Small molecule	Oral	Nrf2 activation and immunomodulation	Advantages: Oral administration; established efficacy Limitations: GI adverse effects, flushing, lymphopenia	(412, 413)
Natalizumab (Tysabri)	α4-integrin monoclonal antibody	IV	Blocks leukocyte trafficking across the BBB	Advantages: Very high efficacy Limitations: Risk of PML, rebound after discontinuation	(401, 402)
Fingolimod (Gilenya)	Small molecule (S1P receptor modulator)	Oral	Functional antagonism of S1P receptors causing lymphocyte sequestration	Advantages: Oral administration; effective relapse reduction Limitations: Lymphopenia, infection risk, rebound disease activity	(409–411)
Diroximel fumarate (Vumerity)	Small molecule	Oral	Nrf2 activation	Advantages: Improved GI tolerability vs. dimethyl fumarate Limitations: Lymphopenia; requires long-term treatment	(423, 424)
Teriflunomide (Aubagio)	Small molecule	Oral	DHODH inhibition, limiting proliferating lymphocytes	Advantages: Convenient oral dosing Limitations: Hepatotoxicity, teratogenicity, relatively modest efficacy	(414–416)
Cladribine (Mavenclad)	Immune reconstitution therapy	Oral	Transient depletion of T and B lymphocytes	Advantages: Short-course treatment Limitations: Prolonged lymphocyte depletion, infection risk	(417, 418)
Glatiramer acetate (Copaxone)	Synthetic peptide	SC	Immune deviation toward anti-inflammatory responses	Advantages: Excellent long-term safety Limitations: Frequent injections; moderate efficacy	(405–407)
Interferon-β (Avonex/Rebif)	Cytokine biologic	IM/SC	Broad immunomodulation	Advantages: Long safety record Limitations: Flu-like symptoms, injection burden, modest efficacy	(403, 404)
BTK inhibitors (e.g., tolebrutinib, fenebrutinib)	Small molecule	Oral	Inhibition of B-cell receptor and microglial signaling	Advantages: Targets both adaptive and innate immunity Limitations: Long-term efficacy and safety under investigation	(419–421)
Anti-IL-17/IL-23 therapies (e.g., secukinumab)	Cytokine monoclonal antibodies	SC	IL-17/IL-23 pathway blockade	Advantages: Selective inhibition of the IL-17/IL-23 axis; effective in Th17-mediated autoimmune diseases. Limitations: Limited efficacy in MS clinical trials	(72, 408)
GSNOR inhibitors (N6022, N91115)	Small molecule	Oral	Elevates endogenous GSNO, enhancing protein S-nitrosylation and selective immune reprogramming	Advantages: Selective suppression of pathogenic Th1/Th17 and inflammatory B cells while promoting Treg/Breg responses; antioxidant and BBB-protective effects; no systemic lymphopenia observed in preclinical models Limitations: Clinical efficacy in MS remains to be established	(53, 80, 81, 133, 134, 163, 219, 228, 422)

Conversely, small-molecule DMTs, such as dimethyl fumarate, diroximel fumarate, fingolimod, siponimod, and teriflunomide, offer several practical advantages, including oral administration, reduced reliance on clinic-based care, and greater dosing flexibility (**Table 1**). Nonetheless, their mechanisms of action, which include sphingosine-1-phosphate (S1P) receptor modulation (e.g., fingolimod and siponimod) and inhibition of pyrimidine synthesis (e.g., teriflunomide), result in broad immunosuppression. These effects are associated with significant clinical risks, including treatment-induced lymphopenia in 30-60% of patients and a two- to three-fold increase in the incidence of serious infections (7, 8). This highlights a critical therapeutic gap: the pressing need for orally available small-molecule agents that can achieve selective immunomodulation without compromising global immune competence.

To address this unmet therapeutic need in MS, we highlight the therapeutic potential of S-nitrosoglutathione (GSNO), a stable intracellular nitric oxide (NO•) carrier, and reversible inhibitors of GSNO reductase (GSNOR), the key enzyme controlling GSNO metabolism. Unlike conventional small-molecule DMTs, these agents act through redox-sensitive signaling pathways, offering selective modulation of autoimmune inflammation, suppression of neuroinflammation, and attenuation of oxidative stress while preserving immune competence. In this review, we summarize current mechanistic and preclinical evidence supporting the GSNO/GSNOR axis as a promising therapeutic target in MS and discuss the translational potential of reversible GSNOR inhibitors, whose favorable safety profiles in patients with asthma and cystic fibrosis support further investigation of their therapeutic efficacy in MS.

This review was conducted as a narrative review rather than a systematic review and therefore did not follow PRISMA guidelines. Relevant literature was identified through searches of PubMed and Google Scholar, supplemented by manual screening of reference lists from key mechanistic, translational, and clinical investigations. Search terms included combinations of “multiple sclerosis,” “experimental autoimmune encephalomyelitis,” “nitric oxide,” “S-nitrosylation,” “S-nitrosoglutathione,” “GSNOR,” “redox,” “oxidative stress,” “inflammation,” “autoimmune disease,” and “lymphocytes.” Over 400 publications from 2000 through June 2026 were considered, along with seminal earlier studies. Priority was given to original research articles, supplemented by selected reviews for conceptual context. Studies were selected based on their mechanistic, translational, or therapeutic relevance to GSNO/GSNOR signaling in MS, EAE, immune regulation, oxidative stress, and neuroinflammation. Conference abstracts, non-English publications, and studies outside the scope of this review were excluded. As a narrative review, the aim was to provide a representative synthesis of the current evidence rather than an exhaustive systematic assessment.

## 2 S-nitrosylation: a stable alternative for NO• signaling regulation under inflammatory and oxidative stress

**Figure 1:**
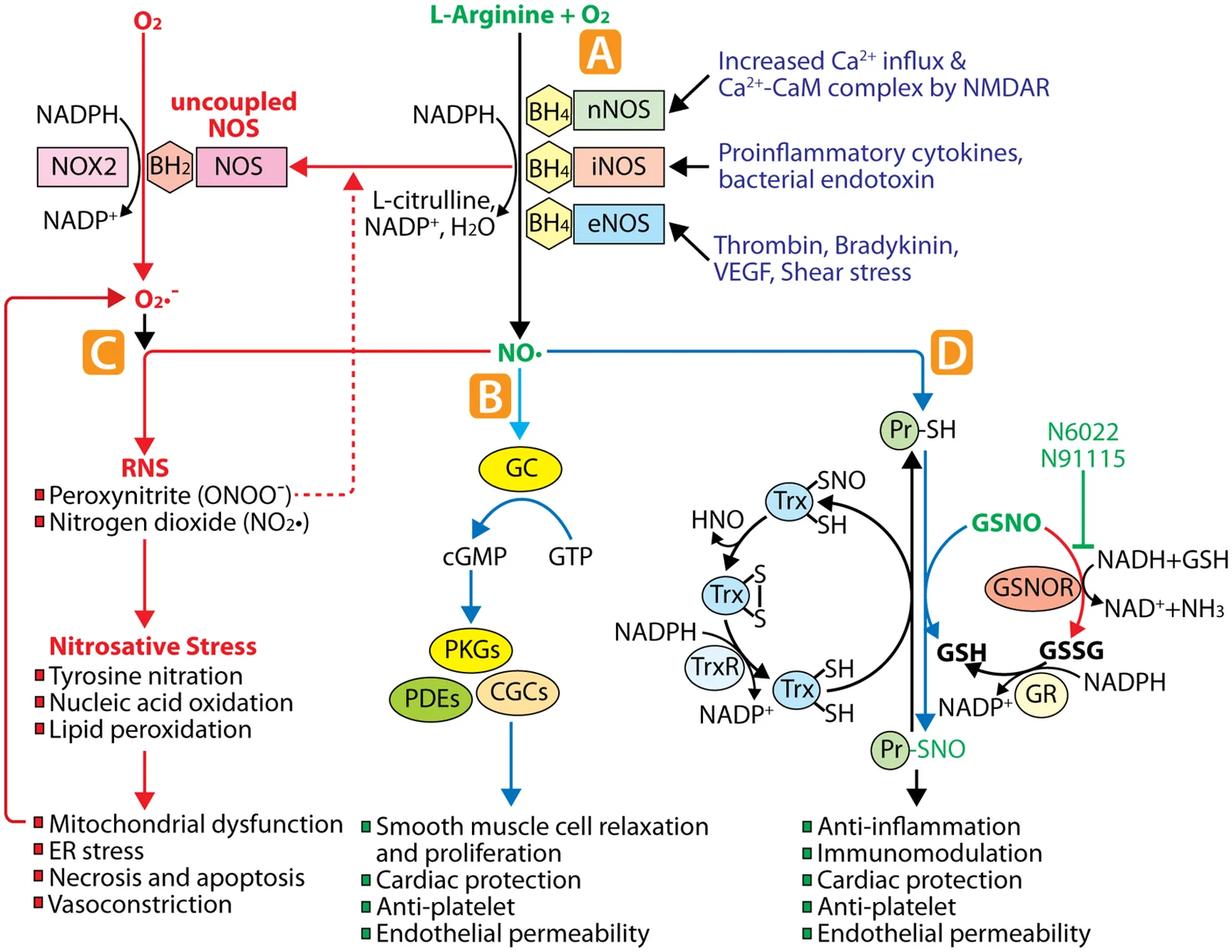
Comprehensive overview of the multifaceted roles of NO• in cellular signaling pathways. **(A)** Nitric oxide (NO•) is synthesized endogenously from L-arginine by three isoforms of nitric oxide synthase (nNOS, eNOS, and iNOS). **(B)** NO• serves as a key signaling molecule by activating soluble guanylyl cyclase (sGC), which catalyzes the production of cyclic GMP (cGMP). cGMP subsequently activates downstream effectors, including cGMP-dependent protein kinases (PKGs), cGMP-gated ion channels (CGCs), and cyclic nucleotide-regulated phosphodiesterases (PDEs), thereby modulating vascular tone, cardiac contractility, and endothelial barrier integrity. **(C)** Under pathological conditions, superoxide anion (O_2_•^-^) is generated by enzymatic sources such as NADPH oxidase (NOX), xanthine oxidase (XO), lipoxygenase (LOX), cyclooxygenase (COX), and mitochondrial dysfunction. O_2_•^-^ reacts rapidly with NO• to form peroxynitrite (ONOO^-^), a potent oxidant that induces tyrosine nitration, nucleic acid oxidation, and lipid peroxidation. ONOO^-^ also oxidizes tetrahydrobiopterin (BH_4_) to dihydrobiopterin (BH_2_), resulting in NOS uncoupling, a pathological switch in NOS activity from NO• generation to O_2_•^-^ production, further reducing NO• bioavailability and impairing NO•-mediated signaling. **(D)** NO• also exerts cellular effects through S-nitrosylation, a reversible post-translational modification of target proteins that regulates diverse physiological processes, including inflammation, immune regulation, cardiovascular protection, antithrombotic activity, and endothelial integrity. Protein S-nitrosylation is primarily mediated by S-nitrosoglutathione (GSNO)-mediated trans-S-nitrosylation, in which GSNO is generated by the reaction of NO• with O_2_ to form N_2_O_3_, a potent nitrosating agent, followed by its reaction with glutathione (GSH). The GSNO-dependent S-nitrosylation signaling pathway is tightly regulated by two major systems. First, GSNO reductase (GSNOR) enzymatically reduces GSNO to hydroxylamine and oxidized glutathione (GSSG), which is subsequently recycled to GSH by glutathione reductase (GR), thereby limiting intracellular GSNO levels and downstream S-nitrosylation. Pharmacological inhibition of GSNOR, using agents such as N6022 and N91115, has been proposed to enhance GSNO-mediated signaling in disease contexts. Second, the thioredoxin (Trx)-thioredoxin reductase (TrxR) system catalyzes protein denitrosylation, with Trx transferring the S-nitroso group from proteins to itself to form nitroxyl (HNO), after which Trx is regenerated by TrxR. Together, these regulatory mechanisms maintain redox homeostasis and fine-tune NO•-dependent signaling under both physiological and pathological conditions.

NO• is a gaseous free radical synthesized endogenously by three distinct nitric oxide synthase (NOS) isoforms: neuronal NOS (nNOS or NOS1), endothelial NOS (eNOS or NOS3), and inducible NOS (iNOS or NOS2) (9) (**Figure 1A**). Under physiological conditions, NO• is generated at low concentrations (picomolar to nanomolar range) primarily by the constitutively expressed isoforms nNOS and eNOS, whose activity is tightly regulated by cell signaling pathways. In this context, NO• serves as a critical signaling molecule that mediates homeostatic cellular responses. By binding to the heme moiety of soluble guanylyl cyclase (sGC), NO• activates cyclic GMP (cGMP) production, which subsequently stimulates protein kinase G (PKG), cGMP-gated ion channels (CGCs), and phosphodiesterases (PDEs). Through these downstream effectors, physiological NO• signaling governs fundamental processes, including vascular tone, platelet aggregation, neuronal plasticity, and endothelial function (10–12) (**Figure 1B**).

Under inflammatory conditions, however, iNOS becomes the dominant source of NO•, producing markedly higher, sustained concentrations, often reaching the micromolar range (13–17). NO• is not inherently toxic; however, under oxidative stress it reacts with molecular oxygen (O_2_) and superoxide anion (O_2_•^-^) to generate reactive nitrogen species (RNS), including nitrogen dioxide radical (NO_2_•) and peroxynitrite (ONOO^-^), which induce cytotoxicity through DNA damage, protein nitration, and lipid peroxidation (18) (**Figure 1C**). Although RNS play a crucial role in antimicrobial defense, these mechanisms also contribute to host tissue and cell damage, leading to outcomes such as endoplasmic reticulum stress, mitochondrial dysfunction, cell death, and vascular impairment (19) (**Figure 1C**). RNS also oxidize tetrahydrobiopterin (BH_4_), a critical NOS cofactor, causing enzyme uncoupling that shifts NO• production toward O_2_•^-^ generation, thereby amplifying oxidative stress and worsening pathology (20, 21) (**Figure 1C**). In this context, strategies to elevate NO• levels, whether through enhanced NOS activity or NO•-releasing drugs, often prove ineffective or harmful, as free NO• is rapidly consumed in RNS formation rather than signaling via the canonical cGMP pathway. To address this limitation, recent approaches employ S-nitrosylating agents that bypass free NO• generation, thereby limiting its conversion into ONOO^-^, and instead directly activate NO•-mediated signaling through targeted protein S-nitrosylation.

S-nitrosylation is a reversible post-translational modification involving the covalent attachment of a NO• group to the thiol group of specific cysteine residues in proteins (22) (**Figure 1D**). To date, more than 20,000 S-nitrosylation sites have been identified across over 10,000 proteins (23), influencing protein function, stability, interactions, and subcellular localization across a wide range of species and functional classes (24). This modification has been implicated in various physiological processes, including neurotransmission, anti-inflammatory responses, immune modulation, and the regulation of vascular tone (25–29).

Currently, the mechanisms by which NO• mediates S-nitrosylation of intracellular proteins are not fully understood. However, growing evidence suggests that NO• does not typically react directly with protein thiols, except under specific conditions. Instead, S-nitrosylation usually occurs indirectly via trans-S-nitrosylation, wherein NO• first reacts with low-molecular-mass thiols such as glutathione (GSH), cysteine, or coenzyme A (CoA) to form low-molecular-mass S-nitrosothiol (LMMSNO) intermediates like GSNO, S-nitrosocysteine, and S-nitroso-CoA (30, 31). These intermediates subsequently transfer the nitroso group to protein thiols via trans-S-nitrosylation.

Among LMMSNOs, GSNO is the most abundant and serves as the principal physiological donor for protein S-nitrosylation (**Figure 1D**). Within cells, GSNO exists in equilibrium with protein S-nitrosothiols (PrSNOs) (32). Therefore, maintaining cellular GSNO homeostasis, tightly regulated by both its synthesis and enzymatic degradation, is essential for the proper control of S-nitrosylation-dependent pathways. GSNO is produced through the reaction of NO• with GSH, the most abundant intracellular reductant; however, this reaction is inefficient in aqueous environments, where direct NO•-GSH interactions typically yield oxidized glutathione (GSSG) and nitroxyl (NO^-^) rather than GSNO (33, 34). Instead, GSNO formation is favored in hydrophobic compartments such as cellular membranes, where NO• and O_2_ accumulate and react to form dinitrogen trioxide (N_2_O_3_), a potent S-nitrosylating intermediate that subsequently reacts with GSH to generate GSNO (35–37). This process is highly sensitive to the intracellular redox state. Notably, under inflammatory and oxidative stress conditions, large amounts of NO• are produced by iNOS, yet concomitantly elevated O_2_•^-^ reacts with NO• at a near diffusion-limited rate (6.7 × 10^9^ M^−1^ s^−1^) to form ONOO^-^ (38, 39). This reaction occurs orders of magnitude faster than the reaction leading to GSNO synthesis, thereby strongly favoring ONOO^-^ generation over S-nitrosothiol formation (40–43).

While GSNO synthesis is tightly influenced by redox conditions, its intracellular levels are also determined by enzymatic degradation, primarily mediated by S-nitrosoglutathione reductase (GSNOR; also known as alcohol dehydrogenase class III/ADH5; EC 1.1.1.1) (**Figure 1D**) (44). GSNOR is a dimeric, NADH-dependent oxidoreductase that acts on various aliphatic compounds, with GSNO and S-hydroxymethyl glutathione (HMGSH) as preferred substrates. It metabolizes GSNO into an N-hydroxy sulphonamide intermediate (GSNHOH), which reacts with GSH to yield hydroxylamine (NH_2_OH) and oxidized glutathione (GSSG) (45). GSNOR, a cysteine-rich protein, can itself be S-nitrosylated by GSNO, forming a feedback loop that enhances its activity (46). Deletion of the GSNOR gene leads to increased levels of both GSNO and protein S-nitrosylation *in vivo* (47). However, GSNOR does not directly denitrosylate protein S-nitrosothiols; this function is primarily carried out by the thioredoxin (Trx)–thioredoxin reductase (TrxR) system (48) (**Figure 1D**).

The pathological relevance of GSNOR dysregulation was first recognized in asthma and cystic fibrosis, where overexpression or hyperactivity of GSNOR reduces GSNO bioavailability, leading to bronchoconstriction and impaired airway relaxation (49, 50). Subsequently, increased GSNOR expression and activity have also been observed in animal models of traumatic brain injury (51), COVID-19 (52), and MS (EAE) (53). For the treatment of asthma and cystic fibrosis, reversible small-molecule GSNOR inhibitors, such as the injectable or inhalable N6022 and the orally bioavailable N91115, have been developed and evaluated in both preclinical models and Phase I/II clinical trials (54, 55). These compounds demonstrated favorable safety profiles, high tolerability, and no dose-limiting toxicities. However, neither agent met the primary clinical endpoints in trials for asthma or cystic fibrosis. Despite these setbacks, they offer a unique advantage: the ability to selectively enhance intracellular S-nitrosylation, thereby supporting physiological NO•-mediated signaling while avoiding cytotoxic RNS generation and minimizing off-target effects. More recently, the therapeutic potential of these GSNOR inhibitors has been explored in animal models of traumatic brain injury, COVID-19, and MS, where they exhibited significant disease-modifying effects (51–53). Nonetheless, despite these promising preclinical findings, neither compound has advanced to clinical trials for these indications. Importantly, these findings distinguish two different aspects of translational development. While Phase I/II studies in asthma and cystic fibrosis support the clinical feasibility and tolerability of GSNOR inhibition in humans, the therapeutic evidence for MS remains entirely preclinical and is currently limited to mechanistic *in vitro* studies and preclinical studies using EAE models. Accordingly, future clinical studies are needed to determine whether the immunomodulatory and neuroprotective effects described in this review translate into clinical benefit for patients with MS.

In summary, GSNOR inhibition enhances GSNO-mediated S-nitrosylation, providing a more sustained means of modulating NO• signaling than direct NO• delivery. Unlike NO•, which has a half-life of only seconds, GSNO is considerably more stable and can persist for hours depending on the cellular environment (56–59). This greater stability enables more prolonged and spatially distributed redox signaling, whereas free NO• primarily acts near its site of production (60, 61). Moreover, GSNO does not spontaneously release free NO• and therefore does not directly promote ONOO^-^ formation under oxidative conditions, potentially reducing nitrosative injury while preserving physiological NO• bioactivity (62–66). Nevertheless, because GSNO regulates numerous S-nitrosylation-dependent signaling pathways, the biological consequences of GSNOR inhibition are likely to depend on the magnitude and context of GSNO elevation.

## 3 S-nitrosylation-mediated regulation of autoimmune responses in MS and EAE

MS exhibits two distinct inflammatory patterns. In acute and relapsing phases, blood-brain barrier (BBB) disruption permits widespread infiltration of T cells and B cells into white matter, forming active demyelinating plaques with elevated inflammatory cytokines (67, 68). In contrast, progressive stages involve gradual lymphocyte accumulation in perivascular and meningeal spaces without major BBB disruption, leading to subpial cortical demyelination and diffuse neurodegeneration in white and gray matter (68). Progressive MS also features ectopic lymphoid follicles (ELFs) in the meninges that are enriched with T and B cells and other immune cells and provide sites for the local production of pro-inflammatory mediators, antigen presentation, and sustained autoimmune responses within the CNS (67). Despite differences in inflammatory patterns between relapsing and progressive stages, both are fundamentally driven by T- and B-cell-mediated autoimmune responses, and we explore how the GSNO-GSNOR axis regulates these responses.

### 3.1 S-nitrosylation-mediated down-regulation of effector CD4^+^ T cells

Studies using experimental autoimmune encephalomyelitis (EAE), an animal model of MS, suggest that CD4^+^ T helper (Th) cells are crucial in triggering the autoimmune-driven inflammatory demyelination (67). This finding aligns with genetic evidence linking MS to MHC class II molecules, which present antigens to these CD4^+^ T cells (69). Among CD4^+^ subsets, Th1 and Th17 cells, which produce IFN-γ and IL-17, respectively, are particularly pathogenic during the early and acute phases of neuroinflammation, and elevated levels of these cytokines correlate with disease activity in patients with MS (67, 70). Clinical evidence further supports their role: IFNγ administration exacerbates MS, whereas IL-17 blockade reduces lesion formation (71, 72).

Th1 and Th17 cells play distinct roles in the pathogenesis of MS and EAE. Th1-derived IFN-γ and TNF-α activate macrophages and microglia and upregulate MHC class II expression on CNS-resident cells, thereby amplifying local inflammation, demyelination, and axonal damage (73). However, the effects of IFN-γ are stage- and cell-dependent. Although IFN-γ generally promotes inflammation during the acute phase, protective effects have been reported during later or chronic phases of EAE (74, 75). IFN-γ also exerts heterogeneous effects on CNS-resident cells, including astrocytes, oligodendrocytes, and microglia, which may partly explain discrepancies among experimental studies. For example, astrocyte-specific IFN-γ signaling was recently shown to limit chronic CNS inflammation through PD-L1 induction (76). These findings indicate that, although Th1-derived IFN-γ contributes substantially to acute neuroinflammation, its overall effects may vary according to disease stage and the responding CNS cell type. In comparison, Th17 cells are key drivers of sustained neuroinflammation through the production of IL-17A, IL-17F, IL-21, and IL-22, which promote BBB disruption, immune-cell infiltration, and demyelination (77). Th17 cells also contribute to the formation of ELFs within the CNS in MS and EAE and are critically involved in the pathogenesis of progressive MS (78).

NO• was initially reported to induce Th1 differentiation (79). Under *in vitro* culture conditions, low concentrations (<10 μM) of NO• donors such as DETA-NO enhance Th1 cell differentiation by activating cGMP-dependent signaling pathways, which in turn upregulate the IL-12 receptor β2 subunit (IL12RB2), a critical mediator of Th1 polarization and effector function (79). In contrast to these effects, GSNO showed no observable impact on Th1 responses *in vitro*. Specifically, Nath et al. demonstrated that treatment of cultured T cells isolated from EAE mice with GSNO at concentrations up to 100 μM did not affect IL-12-induced Th1 polarization, as evidenced by unchanged levels of STAT4 phosphorylation, T-bet expression, and IFN-γ production (80). These findings suggest that, unlike NO• donors, GSNO does not directly modulate Th1 responses under *in vitro* conditions. However, *in vivo* studies revealed a markedly different outcome. Systemic administration of the GSNOR inhibitor N6022 significantly reduced Th1 cell differentiation, expansion, and IFN-γ production in both the spleen and spinal cord of EAE mice (81), an effect similarly observed in GSNOR-deficient mice (53).

**Figure 2:**
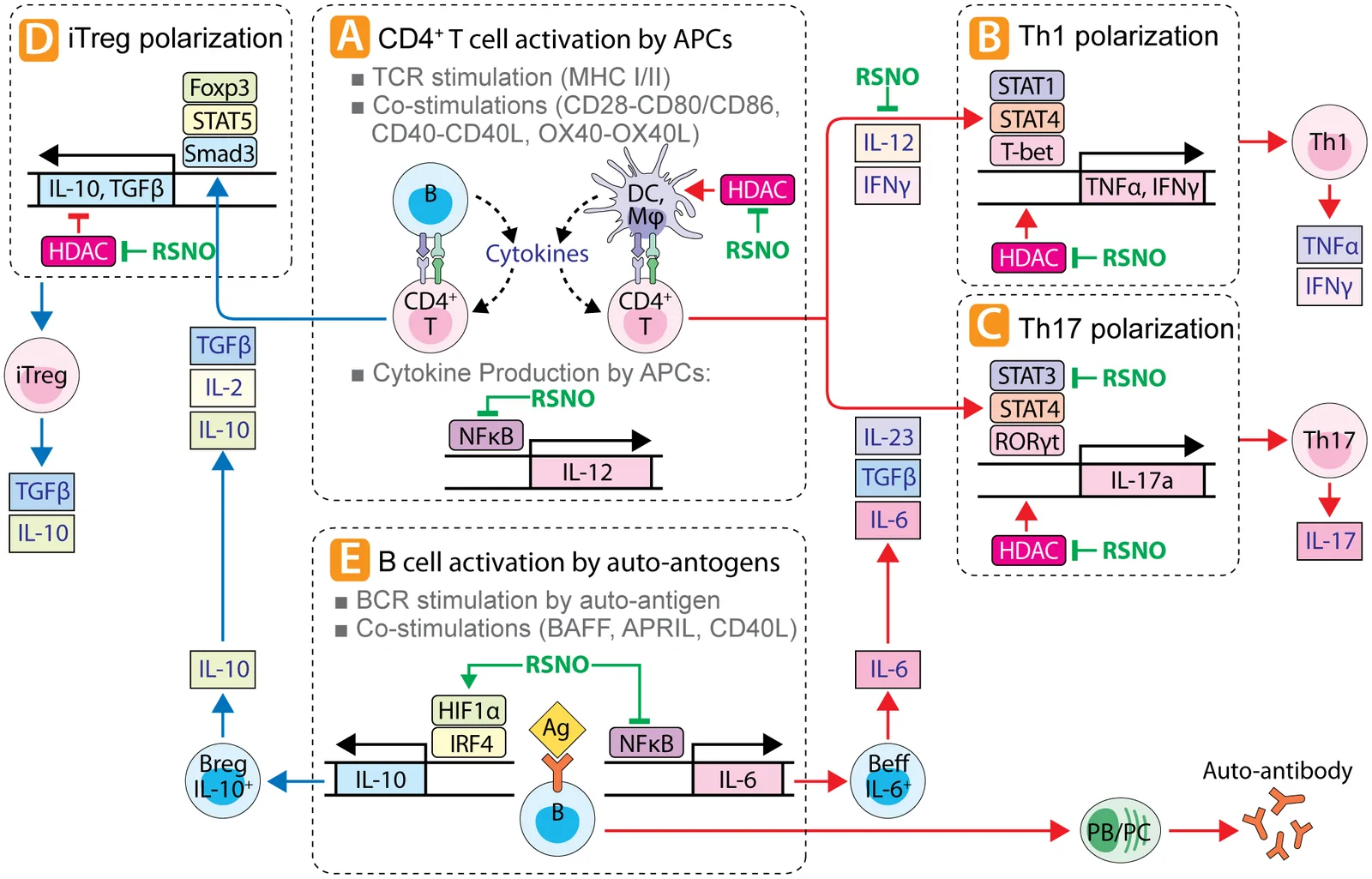
S-nitrosylation-mediated regulation of autoimmune responses in MS and EAE. **(A)** Antigen-presenting cells (APCs), including dendritic cells (DCs), macrophages (Mϕ), and B cells, activate CD4^+^ T cells through TCR stimulation (via MHC I/II) and co-stimulatory signals (CD28-CD80/86, CD40-CD40L, OX40-OX40L), initiating downstream effector differentiation. **(B)** Th1 polarization is driven by IL-12 and IFNγ through STAT1/STAT4 and T-bet signaling. S-nitrosylation (RSNO) suppresses this axis by inhibiting NF-κB activation and IL-12 production in DCs and Mϕ, thereby reducing Th1 differentiation and effector cytokines (IFNγ, TNFα). **(C)** Th17 differentiation is promoted by IL-6, IL-23, and TGF-β via STAT3 and RORγt. RSNO attenuates this process by inhibiting STAT3 phosphorylation and modulating histone deacetylase (HDAC) activity, leading to reduced IL-17 production. **(D)** Induced regulatory T cell (iTreg) differentiation is driven by IL-10, TGF-β, and IL-2 through Smad3 and STAT5 signaling, promoting Foxp3 expression. RSNO enhances iTreg polarization and upregulates IL-10 and TGF-β by suppressing HDAC activity. **(E)** B cell activation via autoantigen-triggered B cell receptor (BCR) signaling and co-stimulation (BAFF, APRIL, CD40L) promotes differentiation into IL-6-producing effector B cells (Beffs) and IL-10-secreting regulatory B cells (Bregs). RSNO skews this balance toward a regulatory phenotype by suppressing IL-6 and enhancing IL-10 production through induction of HIF-1α and IRF4 and inhibition of NF-κB, thereby promoting Treg responses and suppressing Th1 and Th17-mediated inflammation.

The precise mechanisms by which GSNOR inhibition suppresses Th1 immune responses *in vivo* remain incompletely understood. However, findings that S-nitrosylating agents inhibit LPS- and IFN-γ-induced IL-12 production, an essential factor for Th1 differentiation, by inhibiting NF-κB (nuclear factor kappa-light-chain-enhancer of activated B cell) activation in macrophages and dendritic cells (DCs) (82, 83) suggest that GSNOR inhibitors or S-nitrosylating agents may indirectly regulate Th1 differentiation by modulating IL-12 expression in these antigen-presenting cells (**Figures 2A, B**). Consistent with this proposed mechanism, iNOS-deficient mice exhibit exaggerated Th1 responses characterized by elevated IFN-γ production following infections, such as Leishmania major (84), Staphylococcus aureus (85), Herpes simplex virus (86), or in autoimmune models like EAE (87). Together, these studies indicate that low concentrations of NO• promote Th1 differentiation through cGMP-dependent upregulation of the IL-12 receptor, whereas higher levels, through the formation of S-nitrosothiols such as GSNO, suppress Th1 responses indirectly by inhibiting IL-12 production in antigen-presenting cells.

In addition to their effects on Th1 responses, NO• and S-nitrosylating agents also exert regulatory effects on Th17 responses. The regulatory role of NO• and S-nitrosylating agents in Th17 immune responses is supported by evidence showing that iNOS-deficient CD4^+^ T cells exhibit enhanced Th17 differentiation under Th17-polarizing conditions compared to wild-type cells (88). Consistently, iNOS-deficient EAE mice exhibited elevated Th17 cell numbers and IL-17A production relative to wild-type mice, indicating a suppressive role for iNOS-derived NO• in Th17-mediated inflammation (89). Similarly, treatment of wild-type EAE mice with either GSNO or the GSNOR inhibitors (N6022 and N91115) significantly suppressed Th17 responses, improved clinical scores, and reduced spinal cord demyelination (53, 80, 81). Furthermore, similar effects were also observed in GSNOR-deficient EAE mice (53). Most importantly, the inhibition of Th17 as well as Th1 responses by treatment with GSNOR inhibitors did not cause systemic lymphopenia, unlike fingolimod (FTY720) (53, 81), which exerts its therapeutic effect by preventing the egress of lymphocytes from secondary lymphoid organs and thereby reducing their circulation in the periphery (90). This distinction highlights the potential advantage of GSNOR inhibitors in modulating pathogenic T cell responses without broadly suppressing the immune system.

Although the precise mechanism by which GSNO suppresses Th17 immune responses remains to be fully elucidated, evidence indicates that GSNO can inhibit TGF-β- and IL-6/IL-23-driven expression of RORγt by blocking STAT3 activation (phosphorylation) (80) (**Figure 2C**). Supporting this, Kim et al. reported that GSNO suppresses IL-6-induced STAT3 activation via S-nitrosylation at Cys259 of STAT3 (91), indicating a role for S-nitrosylation-dependent signaling in NO•-mediated Th17 suppression. Additionally, NO• has been shown to interfere with TGF-β signaling by inhibiting Smad2/3 phosphorylation and nuclear translocation, via activation of cGMP-dependent PKG-1 (92). Moreover, NO• is known to inhibit IL-17 promoter activity through tyrosine nitration of RORγt (88), suggesting that multiple mechanisms, including S-nitrosylation, nitration, and the cGMP/PKG signaling pathway, collectively contribute to the NO•-dependent regulation of Th17 responses.

### 3.2 S-nitrosylation-mediated upregulation of regulatory CD4^+^ T cells

Regulatory T cells (Tregs) are vital for preserving immune homeostasis and play a pivotal role in suppressing autoreactive immune responses in MS (93–95). In patients with MS, peripheral Treg frequency is generally preserved; however, their immunosuppressive function and migratory capacity to the CNS are markedly impaired. This is reflected by their reduced abundance in brain tissue and enrichment of a highly apoptosis-prone subset in the cerebrospinal fluid, suggesting selective depletion within inflammatory CNS lesions (96, 97). Both numerical and functional deficiencies of Tregs contribute to MS pathogenesis, making them a high-priority therapeutic target for restoring immune tolerance and controlling disease progression (98).

Two main Treg subsets have been described: thymus-derived natural Tregs (nTregs; CD4^+^CD25^+^Foxp3^+^, where Foxp3 is a lineage-defining transcription factor essential for Treg development and suppressive function), which are relatively stable and less influenced by the cytokine milieu, and peripherally induced Tregs (iTregs), which differentiate from naïve CD4^+^ T cells in response to antigenic stimulation and local inflammatory cues (99). iTregs are particularly important in MS as they arise at sites of inflammation and can suppress autoreactive T cells that evade central tolerance (94). Several iTreg subsets exist, including Foxp3^+^ iTregs with high CD25 expression (100); Tr1 cells, which produce IL-10 and TGF-β but lack Foxp3 (101, 102); and Th3 cells, which predominantly secrete TGF-β (103).

Emerging evidence highlights a regulatory role for NO• in the differentiation of peripheral Foxp3^+^ iTreg cells. *In vitro* cell culture experiments have demonstrated that NO• at low concentrations negatively regulates the generation of Foxp3^+^ iTregs through activation of cGMP-dependent PKG-1, which suppresses TGF-β-induced Smad3 phosphorylation and subsequent Foxp3 gene expression (104, 105). These *in vitro* findings highlight NO• as a potential inhibitor of peripheral iTreg differentiation and suggest a suppressive effect on key regulatory pathways.

In contrast, findings from *in vivo* disease models reveal an opposing role for NO•, underscoring the context-dependent complexity of its immunomodulatory functions. In mouse models of colitis and arthritis, administration of the NO• donor DETA-NO alleviated inflammatory pathology by enhancing IL-10 production (106). Furthermore, genetic deletion of GSNOR or its pharmacological inhibition with N6022 or N91115 led to the expansion of Foxp3^+^ iTreg cells and increased IL-10 expression in both the spleen and the CNS of MOG_35-55_-immunized C57BL/6 EAE mice (53, 81). Notably, NO• has also been shown to induce a distinct subset of iTreg cells, termed NO•-Tregs by Niedbala et al. (106), which express CD25 but lack Foxp3 expression. These cells are phenotypically and functionally distinct from conventional Treg subsets, including nTregs, Foxp3^+^ iTregs, Th3 (TGF-β^+^), and Tr1 (IL-10-dependent) cells, due to their unique characteristics and differentiation requirements (106). Consistently, both GSNO treatment and GSNOR knockout have been reported to increase CD4^+^CD25^+^Foxp3^-^ iTreg cell populations in EAE mice [65, 88]. These findings suggest that GSNO may modulate CNS inflammation in EAE by promoting both IL-10-producing Foxp3^+^ and Foxp3^-^ iTregs, although the underlying molecular mechanisms remain to be elucidated (**Figure 2D**).

### 3.3 Potential involvement of histone deacetylases in S-nitrosylation-mediated regulation of effector vs. regulatory T cells

Histone acetyl transferases (HATs) and histone deacetylases (HDACs) regulate gene expression by catalyzing the acetylation and deacetylation of histone and non-histone proteins (107–109). In MS, HDACs contribute to disease pathogenesis by promoting pro-inflammatory functions of DCs and T cells. Specifically, HDACs enhance DC expression of costimulatory molecules and cytokines such as IL-6, IL-12, and TNF-α, which drive Th1 and Th17 differentiation (110–113) (**Figure 2A**). Simultaneously, multiple studies have reported that HDACs suppress Treg generation and function, compromising immune tolerance (114–120). Specifically, HDACs are known to inhibit the acetylation of Foxp3, thereby reducing its stability and transcriptional activity, ultimately impairing Treg polarization (121) (**Figure 2D**).

In EAE, HDAC inhibitors such as sodium phenylbutyrate and trichostatin A (TSA) attenuate clinical severity and neuropathology by reducing spinal cord inflammation, demyelination, and neuronal loss, while concurrently upregulating neuroprotective genes and downregulating apoptotic markers (122, 123). HDAC inhibitors also impair DC activation and T cell proliferation, in part by inducing the immunoregulatory enzyme IDO (indoleamine 2,3-dioxygenase) (112, 124, 125). Beyond immune modulation, HDAC inhibitors also promote neuronal survival by enhancing antioxidant responses and stabilizing axonal structures (126–128). These dual anti-inflammatory and neuroprotective effects support their therapeutic potential in MS.

S-nitrosylation is increasingly recognized as a key post-translational modification that regulates the activity, localization, and interactions of specific HDACs, including HDAC2 (129, 130), HDAC6 (131), and SIRT1 (132), thereby influencing gene expression and diverse cellular functions. For instance, neurotrophin-generated NO• S-nitrosylates HDAC2 at Cys262 and Cys274, disrupting its chromatin binding and thereby promoting histone acetylation, chromatin relaxation, and transcription of genes critical for neuronal development, cardiac function, and neuroprotection (129, 130). Similarly, HDAC6 is S-nitrosylated by iNOS-derived NO• in cytokine-treated cells, leading to reduced deacetylase activity and increased acetylation of target proteins (131), while SIRT1, a Sir2 family HDAC, is inactivated by nNOS-generated NO• through S-nitrosylation of its CXXC motif, causing increased acetylation of target proteins, such as PGC-1α (132). These findings underscore S-nitrosylation as a dynamic and site-specific regulatory mechanism that modulates HDAC function in both physiological and pathological contexts, including those relevant to neuroprotection and immune regulation.

Taken together, both pharmacological inhibition of HDACs and redox-dependent S-nitrosylation modulate HDAC function, ultimately promoting the expression of anti-inflammatory and neuroprotective genes. Notably, HDAC inhibitors have also been reported to suppress Th1 and Th17 immune responses while promoting Treg cell differentiation (110–120), effects that closely resemble those of GSNO and GSNOR inhibitors reported in EAE models (52, 53, 80, 81, 133, 134) (**Figure 2**). Since HDAC activity can be inhibited through S-nitrosylation, future studies should investigate the potential of GSNO treatment and GSNOR inhibition to therapeutically reprogram autoreactive T cells by targeting HDACs, thereby alleviating disease in MS and EAE through both immune regulation and neuroprotection.

### 3.4 S-nitrosylation-mediated regulation of B cells in MS and EAE

B cells play a central role in autoimmunity by presenting autoantigens to T cells and producing autoantibodies following their differentiation into plasma cells (135) (**Figures 2A, E**). In addition, B cells also contribute to autoimmune-driven inflammation by secreting pro-inflammatory cytokines, such as IL-6, IFN-γ, and GM-CSF, that exacerbate immune pathology (135, 136) (**Figure 2E**). Among these, IL-6- producing B cells are particularly important in the pathogenesis of MS, as their expansion in patients promotes Th17 responses, while targeted deletion of IL-6 in B cells has been shown to reduce disease severity in EAE models (137, 138) (**Figure 2E**). These findings support the use of anti-CD20 monoclonal antibodies (mAbs) for B-cell depletion, as demonstrated in EAE models where such treatment selectively reduced IL-6-producing B cells and alleviated disease severity (137). Building on this rationale, anti-CD20 mAb therapies, including ocrelizumab, rituximab, and ofatumumab, have been tested in MS and are now approved for clinical use in both MS and rheumatoid arthritis (139, 140). However, their therapeutic efficacy in MS remains modest, and broad B-cell depletion is associated with notable risks, such as increased susceptibility to opportunistic infections (140, 141). Moreover, in other autoimmune diseases, including systemic lupus erythematosus, ulcerative colitis, and psoriasis, anti-CD20 mAb therapies have shown limited benefits or even adverse outcomes (142–144).

Recent evidence suggests that the limited efficacy of anti-CD20 mAbs in autoimmune diseases may stem from their indiscriminate depletion not only of pathogenic effector B (Beff) cells but also of immunosuppressive regulatory B (Breg) cells, whose loss can further exacerbate autoimmunity (145). Bregs exert their effects primarily through anti-inflammatory cytokines such as IL-10, IL-35, and TGF-β, with IL-10 being the most extensively studied in MS (136, 146). Multiple IL-10-producing Breg cell subsets have been identified, including CD24^hi^ CD27^hi^ B cells and CD24^hi^ CD38^hi^ immature B cells in humans (147, 148) and CD1d^hi^ CD5^+^ B cells in mice (149). IL-10-producing Breg cells are known to suppress Th1 and Th17 responses, inhibit antigen presentation by macrophages and dendritic cells, and promote Treg induction (150–153) (**Figure 2E**). Consistent with this, IL-10-producing Breg cells have been implicated in the remission and recovery phases of EAE (154, 155). Collectively, these findings highlight the potential drawbacks of pan-B cell depletion with anti-CD20 mAbs and underscore the need for targeted therapies that inhibit effector B cell activity while preserving or enhancing regulatory B cell functions.

Mouse and human B cells are known to express iNOS; however, its role in humoral immunity remains uncertain due to conflicting reports (156, 157). For instance, iNOS-deficient mice display elevated antiviral IgG2a antibodies and enhanced resistance to influenza A virus infection, pointing to a suppressive role of iNOS in systemic antibody responses (158). In contrast, other studies indicate that iNOS deficiency impairs IgA class switching and weakens mucosal defense against pathogens (159). Moreover, iNOS-derived NO• has been shown to promote B cell and plasma cell survival by preventing apoptosis and extending plasma cell longevity through S-nitrosylation-mediated inhibition of caspases (160–162). Collectively, these findings highlight the context-dependent roles of NO•, which can either restrain or support humoral immunity depending on the anatomical site, disease setting, and type of immune challenge.

Although the impact of iNOS-derived NO• and S-nitrosylating agents on humoral immune responses in MS and EAE remains poorly defined, a recent study reported that treatment with the GSNOR inhibitor N6022 after EAE disease onset in a B cell-dependent EAE model (C57BL/6 mice immunized with the recombinant human MOG extracellular domain, rhMOG_1-125_) led to reduced circulating levels of anti-rhMOG_1–125_ autoantibodies (163). These observations suggest that S-nitrosylating agents may modulate pathogenic autoantibody production in EAE, providing a potential therapeutic avenue for targeting humoral immune responses in MS.

Beyond their role in humoral immunity, NO• also regulates B cell cytokine expression and thereby influences CD4^+^ T cell-mediated autoimmune responses. Kim et al. showed that *in vitro* treatment of mouse and human B cells with GSNO (50 μM) reduced IL-6 and increased IL-10 expression following BCR activation with anti-IgM and co-stimulation with CD40 ligand (or anti-CD40 mAb) and recombinant BAFF (133). Consistent results were observed in EAE mice immunized with either MOG_35-55_ (a CD4^+^ T cell-dependent EAE) or rhMOG_1-125_ (a model dependent on both CD4^+^ T cells and B cells), where treatment with GSNO or the GSNOR inhibitor N6022 upregulated IL-10 and downregulated IL-6 expression in B cells from both the spleen and spinal cord (133, 163). Notably, GSNO treatment of cultured B cells and N6022 treatment in EAE mice enhanced IL-10 and suppressed IL-6 expression not only in CD1d^hi^ CD5^+^ regulatory B cells (Bregs or B10 cells) but also across a broad range of peripheral B cell subsets, including B1a, B1b, transitional (T1 and T2), marginal zone (MZ) and MZ precursor cells, memory B cells, plasmablasts, and plasma cells (133). Furthermore, transfer of B cells from N6022-treated or GSNOR-deficient EAE mice alleviated disease severity in recipient mice with active EAE (133). This therapeutic benefit was associated with elevated IL-10, reduced IL-6, expansion of iTreg cells, and a reduction in Th17 cells in the recipients. Together, these findings indicate that B cells from N6022-treated or GSNOR-deficient EAE mice mitigate disease by reprogramming CD4^+^ T cell-mediated autoimmune responses.

Although the mechanism by which GSNO-mediated S-nitrosylation selectively regulates IL-10 and IL-6 in B cells remains unclear, hypoxia-inducible factor-1 (HIF-1) appears to play a role. HIF-1, composed of a constitutive HIF-1β subunit and an oxygen-sensitive HIF-1α subunit, is a central regulator of metabolic gene expression under hypoxic and inflammatory conditions (164, 165). Under normoxic conditions, HIF-1α is rapidly degraded through PHD2 (prolyl hydroxylase domain protein 2)-dependent proline hydroxylation, followed by pVHL (von Hippel–Lindau protein, a component of the E3 ubiquitin ligase complex)-mediated ubiquitination and proteasomal degradation (166–169). By contrast, under hypoxic conditions, PHD2 activity is inhibited, allowing HIF-1α to escape degradation, accumulate, and translocate to the nucleus where it dimerizes with HIF-1β to activate target gene transcription.

In a murine model of colitis, HIF-1 has been shown to suppress IL-6 and IL-23 production in dendritic cells (170), while promoting IL-10 production by macrophages under hypoxic conditions (171). Activated B cells also utilize this transcription factor to enhance IL-10 expression. Meng et al. demonstrated that HIF-1α directly binds to the hypoxia-responsive element (HRE) in the IL-10 promoter, thereby increasing its transcription in B cells (172). In addition, conditional deletion of HIF-1α in B cells led to reduced IL-10 levels and decreased CD1d^hi^CD5^+^ Breg cell numbers, while aggravating Th17-mediated inflammation in models of arthritis and EAE (172).

Interestingly, NO• has been reported to stabilize HIF-1α via S-nitrosylation of HIF-1α itself, as well as of PHD2 and pVHL, thereby inhibiting proline hydroxylation and subsequent ubiquitination and degradation even under normoxic conditions (173–175). These findings suggest that GSNO, by promoting S-nitrosylation, may enhance HIF-1α stabilization in B cells, leading to increased binding to the IL-10 promoter and amplification of hypoxia-responsive transcriptional programs (**Figure 2E**).

Interferon Regulatory Factor 4 (IRF4) is a transcription factor essential for multiple aspects of the humoral immune response, including B cell differentiation and cytokine production (176–179). It has been shown to enhance IL-10 expression in B cells while suppressing IL-6 production and Th17-mediated immune responses (180–183). In a mouse model of EAE, B cell-specific IRF4 conditional knockout resulted in the absence of IL-10-producing plasmablasts in draining lymph nodes and led to exacerbated disease severity (180). NO• has been reported to induce IRF4 expression in T cells (184), where it negatively regulates proinflammatory gene expression (183). Consistently, Kim et al. found that GSNO treatment enhanced IL-10 and suppressed IL-6 expression in activated B cells through upregulation of both HIF-1α and IRF4 (133). Mechanistically, IRF4 is induced in B cells via BCR-mediated activation of phosphoinositide 3-kinase (PI3K) signaling (185), and GSNO is known to promote PI3K activation by S-nitrosylating and inhibiting its negative regulator, PTEN (phosphatase and tensin homolog) (186). Additionally, GSNO activates c-Src kinase (187), which phosphorylates and activates IRF4 (188). These findings suggest a potential role for IRF4 in GSNO-mediated induction of Breg cells and IL-10 expression in B cells (**Figure 2E**).

Taken together, these findings demonstrate that S-nitrosylation, mediated by iNOS-derived NO• and GSNO, exerts context-dependent regulatory effects on B cells in MS and EAE by modulating their survival, cytokine profiles, and functional phenotypes. Through HIF-1α and IRF4 signaling, S-nitrosylation may contribute to the enhancement of IL-10 and suppression of IL-6 expression, thereby promoting a regulatory B cell phenotype. These immunomodulatory effects not only provide mechanistic insight into the limited efficacy of current pan-B cell depletion therapies, such as anti-CD20 monoclonal antibodies, but also underscore the therapeutic potential of redox-targeted interventions that selectively suppress pathogenic B cells while preserving or boosting regulatory B cell function in MS and EAE.

### 3.5 Potential roles of GSNO-mediated S-nitrosylation in emerging immune cell populations in MS and EAE

Although CD4^+^ T cells and B cells are the major immune populations driving MS pathogenesis, increasing evidence indicates that additional immune cell subsets, including CD8^+^ T cells, natural killer (NK) cells, and Th9 cells, also contribute to disease progression. Compared with conventional CD4^+^ T cells and B cells, relatively little is known about the role of GSNO in regulating these immune populations. Nevertheless, numerous studies have demonstrated that NO• modulates their differentiation, activation, and effector functions through redox-dependent mechanisms, suggesting that GSNO-mediated S-nitrosylation may similarly influence their biological activities.

CD8^+^ T cells are now recognized as important contributors to disease pathogenesis. They are abundant in active MS lesions and directly damage oligodendrocytes and neurons through perforin- and granzyme-mediated cytotoxicity, as well as amplify CNS inflammation by producing IFN-γ and TNF-α (189). Importantly, accumulating evidence indicates that distinct CD8^+^ T-cell subsets possess immunoregulatory activity capable of suppressing autoreactive immune responses, underscoring the dual pathogenic and regulatory roles of CD8^+^ T cells in MS (189). At present, whether GSNO regulates CD8^+^ T cells in MS or EAE remains poorly understood. However, NO• promotes CD8^+^ T-cell survival through reversible S-nitrosylation of caspases, thereby inhibiting activation-induced apoptosis (190). At low concentrations, NO• also enhances CD8^+^ T-cell differentiation and effector function by upregulating IL-12Rβ2 through the sGC/cGMP pathway, thereby increasing IL-12 responsiveness and promoting Tc1 differentiation and IFN-γ production (191, 192). However, at high concentrations, NO• suppresses these CD8^+^ T-cell responses (191, 192). Evidence from a viral lung inflammation model further indicates that GSNO or pharmacological GSNOR inhibitor treatment reduces the infiltration of hyperactivated cytotoxic CD8^+^ T cells and attenuates tissue inflammation (52). Although these findings suggest a potential role for GSNO in regulating pathogenic CD8^+^ T-cell responses, whether similar mechanisms operate during autoimmune neuroinflammation in MS or EAE remains unknown and warrants further investigation.

NK cells are increasingly recognized as important regulators of autoimmune neuroinflammation through their interactions with T cells, B cells, dendritic cells, and microglia (193, 194). Human NK cells are broadly divided into CD56^bright^ and CD56^dim^ subsets (195). CD56^bright^ NK cells primarily exert immunoregulatory functions by eliminating autoreactive CD4^+^ T cells as well as immature dendritic cells, thereby limiting Th1- and Th17-mediated inflammation (196, 197). In contrast, CD56^dim^ NK cells represent the major circulating NK-cell population and exhibit potent cytotoxic activity through perforin- and granzyme-mediated killing (198). Consistent with these functional differences, reduced numbers or impaired function of CD56^bright^ NK cells are associated with increased MS disease activity, whereas excessive activation of CD56^dim^ NK cells may further amplify CNS inflammation under pathological conditions (196, 199). Although direct evidence for GSNO-mediated regulation of NK cells is limited, the effects of NO• on NK-cell biology have been investigated more extensively. High levels of NO• impair NK-cell function by inducing mitochondrial dysfunction, characterized by loss of mitochondrial membrane potential, increased intracellular ROS levels, and apoptosis (200). Interestingly, CD56^bright^ NK cells possess greater antioxidant capacity and exhibit increased resistance to oxidative stress than CD56^dim^ NK cells (201). These findings suggest that the two NK-cell subsets may respond differently to excessive iNOS-derived NO• during neuroinflammation. CD56^dim^ NK cells may be more susceptible to NO•-induced oxidative stress, whereas CD56^bright^ NK cells may be relatively resistant because of their greater antioxidant capacity. Further studies are needed to determine how GSNO-mediated S-nitrosylation affects the survival, activation, and effector functions of these NK-cell subsets in MS and EAE.

Th9 cells are an emerging CD4^+^ T-cell subset implicated in MS pathogenesis. Increased frequencies of Th9 cells and elevated IL-9 levels are reported in patients with MS and in animals with EAE (202). Although initially associated with allergic inflammation, IL-9 is now recognized as an important regulator of autoimmune neuroinflammation. In EAE, IL-9 is reported to enhance the suppressive activity of Foxp3^+^ Treg cells while modulating Th17 responses, suggesting that Th9 cells function as immunomodulators rather than primary drivers of disease (203). Consistent with this concept, higher levels of IL-9 in the cerebrospinal fluid are associated with lower inflammatory activity and reduced disability progression in relapsing-remitting MS (202). Although GSNO-mediated regulation of Th9 cells remains largely unexplored, NO• is reported to promote Th9 differentiation through Mdm2-dependent S-nitrosylation, leading to activation of the p53–IL-2–STAT5–IRF4 signaling pathway (184). NO• is also reported to enhance IL-9 expression through HIF-1α-dependent metabolic reprogramming (204). Interestingly, HIF-1α and IRF4 are also involved in GSNO-mediated regulation of regulatory B cells (133), thus raising the possibility that similar redox-dependent mechanisms contribute to Th9 differentiation. Further studies are likely to delineate how GSNO-mediated S-nitrosylation regulates Th9-cell development and function in MS and EAE.

Collectively, accumulating evidence indicates that NO• regulates multiple immune cell populations beyond conventional CD4^+^ T cells and B cells, including CD8^+^ T cells, NK cells, and Th9 cells. Although direct evidence for GSNO-mediated S-nitrosylation in these immune subsets remains limited, the well-established role of NO• in controlling their activation, differentiation, and effector functions suggests that GSNO may similarly regulate these processes through shared redox-sensitive signaling pathways. Elucidating how GSNO influences these emerging immune populations should improve our understanding of immune regulation in MS and EAE and may also uncover additional mechanisms underlying the therapeutic benefits of GSNO and GSNOR inhibition.

## 4 S-nitrosylation as a regulator of vascular and CNS inflammation in MS and EAE

In MS and EAE, disruption of BBB integrity is an early and pivotal event that permits the infiltration of peripheral immune cells into the CNS (205, 206). Among these infiltrating cells, circulating myeloid populations, particularly monocytes and neutrophils, play a central role in initiating and sustaining neuroinflammation. Once inside the CNS, these cells, together with resident microglia, interact with encephalitogenic T and B lymphocytes and differentiate into either proinflammatory (M1) or anti-inflammatory (M2) phenotypes, thereby shaping the local immune milieu and contributing to demyelination, neuronal injury, and overall disease progression (207, 208). During this process, NO•, primarily produced by iNOS, exerts dual, context-dependent effects: while excessive NO• and reactive nitrogen species (RNS) formation can contribute to tissue damage, NO• can also mitigate inflammation by modulating leukocyte infiltration, cytokine signaling, and redox homeostasis (32, 209). This section discusses the multifaceted roles of NO•, particularly through S-nitrosylation-mediated mechanisms, in modulating CNS inflammation in MS and EAE, with a focus on its impact on vascular integrity, immune cell infiltration and function, myeloid cell polarization, and regulation of proinflammatory gene expression.

### 4.1 S-nitrosylation-mediated regulation of vascular inflammation in MS and EAE

In MS, increased BBB permeability is a common pathological feature observed across different disease subtypes, including RR-MS, SP-MS, and PP-MS (210, 211). BBB disruption is particularly prominent during active inflammatory phases and facilitates the entry of peripheral immune cells and blood-derived molecules into the CNS, contributing to sustained neuroinflammation and neurodegeneration (212). BBB breakdown is a dynamic and multifactorial process involving hemostatic imbalance (e.g., thrombin activation), oxidative stress, aberrant angiogenesis, and inflammatory signaling (212). Notably, emerging evidence indicates that early BBB disruption is at least partially reversible, suggesting that it may serve as a therapeutic window for early intervention (212). Early BBB damage is triggered by cytoskeletal changes involving the formation of F-actin stress fibers, which lead to endothelial cell deformation. Mechanistically, this process is driven by aberrant activation of RhoA, resulting in sustained phosphorylation of endothelial myosin light chain (MLC) (213). These alterations destabilize intercellular junctions, ultimately leading to the disassembly and matrix metalloproteinase (MMP)-mediated degradation of tight junction components, including occludin, claudin, JAMs, and ZO-1, which are hallmark features of early BBB disruption (214–216).

**Figure 3:**
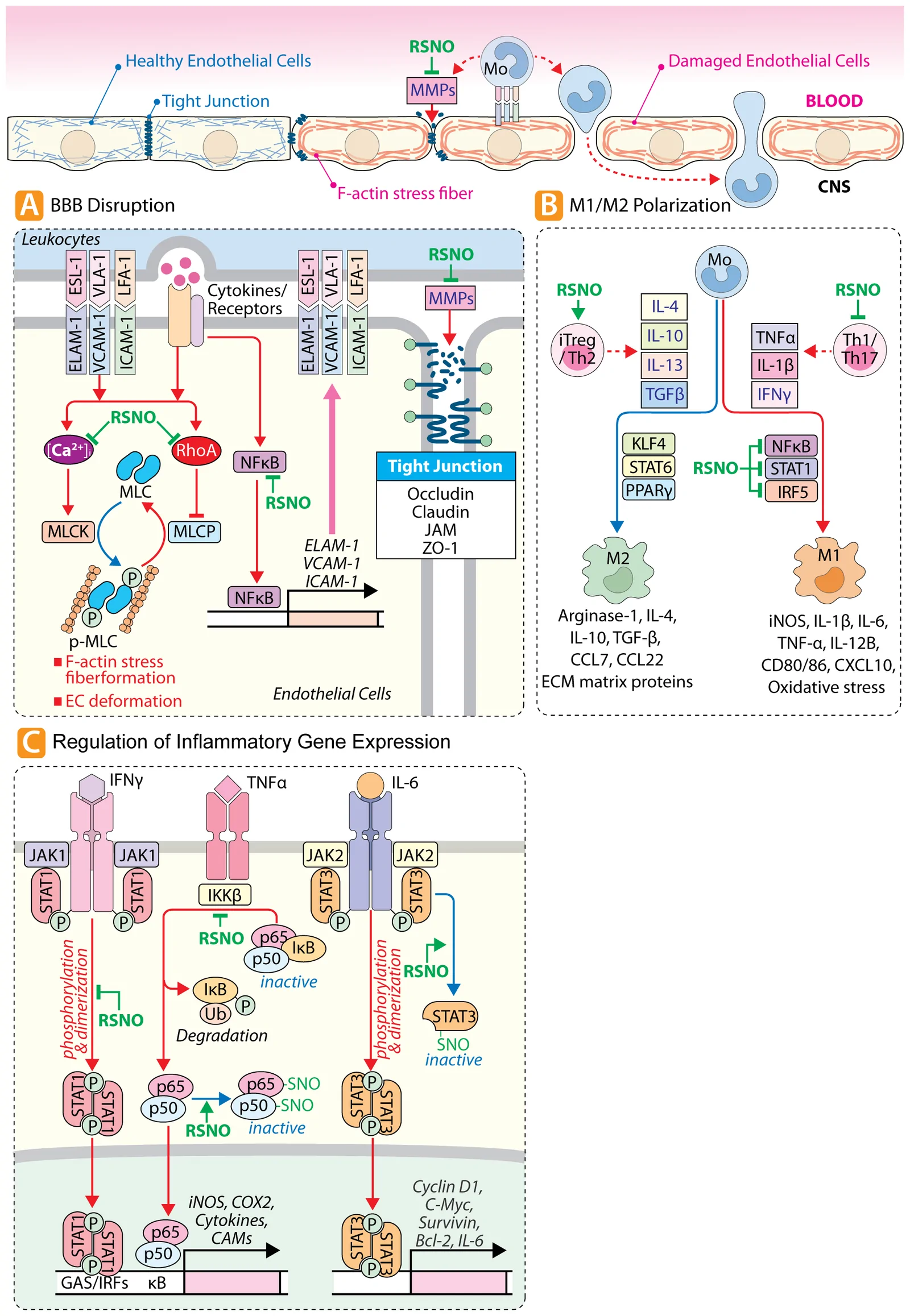
Regulation of CNS vascular and neuroinflammation by S-nitrosylation in MS and EAE. **(A)** S-nitrosylation preserves blood-brain barrier (BBB) integrity. In MS and EAE, BBB disruption results from RhoA-mediated cytoskeletal remodeling (F-actin stress fiber formation) and endothelial cell (EC) deformation and MMP-induced degradation of tight junction proteins (occludin, claudin, JAM, ZO-1). S-nitrosylating agents (RSNO), including GSNO, attenuate EC deformation and barrier disruption by inhibiting RhoA activity and suppressing intracellular Ca²^+^ influx, thus preventing phosphorylation of myosin light chain (MLC), leading to F-actin stress fiber formation, and inhibiting MMP activity. RSNO also suppresses endothelial cell-leukocyte adhesion and infiltration. Inflammatory stimuli upregulate endothelial cell adhesion molecules, such as vascular cell adhesion molecule-1(VCAM-1), intercellular adhesion molecule-1 (ICAM-1), and E-selectin (ELAM-1), facilitating immune cell infiltration into the CNS. GSNO reduces TNFα-induced CAM expression by inhibiting NF-κB activation through S-nitrosylation of its p65/p50 subunits and upstream kinase IKKβ. This limits leukocyte adhesion and transmigration across the BBB, dampening CNS inflammation. **(B)** RSNO via S-nitrosylation modulates macrophage/microglia polarization and inflammatory gene expression. Within the CNS, myeloid cells adopt proinflammatory M1 or anti-inflammatory M2 phenotypes in response to cytokine cues. RSNO inhibits M1 polarization by suppressing NF-κB, STAT1, and IRF5 signaling, while indirectly promoting M2-associated pathways (STAT6, PPARγ, KLF4) through increased Th2/iTreg cytokines (IL-4, IL-13, IL-10). **(C)** RSNO also modulates proinflammatory gene expression by S-nitrosylating transcription factors such as NF-κB, STAT1, and STAT3. This modification leads to the downregulation of proinflammatory mediators, including iNOS, cytokines, and CAMs, suppresses leukocyte proliferation, and reduces IL-6 expression, thereby attenuating neuroinflammation and promoting immune resolution.

Studies have shown that eNOS-derived NO• plays a critical role in regulating endothelial cytoskeletal dynamics (217), but its effects vary depending on the surrounding redox environment. Under oxidative stress, NO• reacts with O_2_•^-^ to form ONOO^-^, which activates RhoA via tyrosine N-nitration, promoting stress fiber formation and compromising the endothelial barrier (218, 219). On the other hand, NO•, via the formation of GSNO, inhibits these processes through S-nitrosylation-mediated inhibition of RhoA (219, 220) (**Figure 3A**). Kim et al. (219) reported that, in human brain endothelial cell cultures, GSNO treatment inhibited thrombin-induced F-actin stress fiber formation and endothelial barrier disruption by suppressing RhoA activation, thereby preserving MLC phosphatase (MLCP)-mediated MLC dephosphorylation (221), and by reducing intracellular Ca^2+^ influx, which in turn limited activation of MLC kinase (MLCK) (222) (**Figure 3A**). Accordingly, GSNO treatment in EAE mice reduced BBB disruption, as indicated by decreased Evans blue dye extravasation into the CNS, and alleviated both inflammatory demyelination and clinical symptoms of EAE (219). These findings highlight the role of GSNO- mediated S-nitrosylation in maintaining BBB integrity throughout the course of MS and EAE.

Beyond structural disruption of the endothelial barrier, infiltration of peripheral immune cells into the CNS requires active engagement with the endothelium via cell adhesion molecules (CAMs) (223). In MS and EAE, pro-inflammatory mediators upregulate endothelial expression of key CAMs such as vascular cell adhesion molecule-1 (VCAM-1), intercellular adhesion molecule-1 (ICAM-1), and E-selectin (or endothelial-leukocyte adhesion molecule-1 or ELAM-1). These molecules interact with their ligands, such as E-selectin ligand-1 (ESL-1), very late antigen-4 (VLA-4; integrin α4β1), and lymphocyte function-associated antigen-1 (LFA-1), on circulating leukocytes, particularly T cells, monocytes, and other myeloid cells, thereby facilitating firm adhesion and migration into the CNS parenchyma (224, 225) (**Figure 3A**). Importantly, the temporal and spatial expression of these adhesion molecules correlates with leukocyte infiltration during disease progression (226). In fact, their increased expression often appears before any clinical symptoms develop in EAE, highlighting their key role in triggering CNS inflammation (227).

Previously, Prasad et al. (228) reported that GSNO treatment of brain endothelial cells *in vitro* reduced TNF-α-induced expression of ICAM-1, VCAM-1, and E-selectin, through S-nitrosylation-mediated inhibition of NF-κB p65. They proposed that TNF-α stimulation leads to denitrosylation of S-nitrosylated p65 in endothelial cells, thereby allowing the p65/p50 NF-κB complex to translocate to the nucleus and induce transcription of CAM genes (**Figures 3A, C**). However, GSNO treatment attenuates this TNFα-induced denitrosylation, limiting NF-κB binding to CAM gene promoters and thereby reducing their transcription (228). The downregulation of CAMs subsequently impairs endothelial cell-leukocyte (e.g., monocyte) interactions, leading to decreased leukocyte infiltration into the CNS in EAE models (228). These findings highlight the therapeutic potential of GSNO in reducing vascular inflammation and immune cell infiltration in MS and EAE.

### 4.2 Macrophage and microglial polarization: M1 vs. M2 in MS and EAE

During the effector phase of EAE, infiltrating monocytes differentiate into macrophages within the CNS, while resident microglia become activated. These changes are largely driven by infiltrating autoreactive T cells, particularly Th1 and Th17 subsets, which secrete proinflammatory cytokines such as TNF-α, IFN-γ, GM-CSF, and IL-17 that modulate the local inflammatory environment and contribute to CNS pathology (229, 230). Within this inflammatory milieu, activated macrophages and microglia predominantly acquire a proinflammatory M1 phenotype. M1 polarization is promoted by cytokines such as TNF-α, IFN-γ, and IL-1β and is regulated by transcription factors including NF-κB, STAT1, and IRF5, which induce the expression of inflammatory mediators such as TNF-α, IL-1β, IL-6, and iNOS (231–235) (**Figure 3B**). M1 macrophages accumulate in demyelinated lesions in MS patients, where they contribute to neuroinflammation and demyelination (236). However, this proinflammatory state is not fixed; myeloid cells retain the capacity to undergo functional reprogramming toward an anti-inflammatory M2 phenotype. This shift is driven by cytokines such as IL-4, IL-13, IL-10, and TGF-β, which activate transcription factors including STAT6, PPARγ, and KLF4 (237–239) (**Figure 3B**). M2 polarization drives the upregulation of genes involved in anti-inflammatory and immunomodulatory responses (e.g., IL-10, TGF-β, CCL7, and CCL22), as well as pathways supporting tissue repair, remyelination, neuroprotection, and metabolic adaptation that promote sustained regulatory function (240–242) (**Figure 3B**).

M1 and M2 phenotypes of macrophages and microglia also differ in their NO• profiles: M1 cells express high levels of iNOS and produce abundant NO•, supporting pathogen and tumor cell elimination, whereas M2 cells express arginase-1, which channels arginine toward ornithine and subsequently proline, a major amino acid in collagen synthesis for tissue repair, while limiting NO• production to prevent further tissue damage (233). Interestingly, accumulating evidence suggests that iNOS-derived NO• may exert negative feedback on M1 polarization (243). In support, splenocytes from iNOS-deficient mice exhibit enhanced M1 macrophage polarization under pro-inflammatory conditions (83). Mechanistically, M1 polarization depends on the activation of transcription factors such as NF-κB and IRF5, which are known to be inhibited by S-nitrosylation and tyrosine nitration, respectively (83, 243) (**Figure 3B**).

Beyond its direct effects on inflammatory signaling pathways, accumulating evidence suggests that NO• also regulates macrophage function through immunometabolic mechanisms. Recent studies have identified metabolic reprogramming as a key regulator of macrophage activation and polarization. In this context, itaconate, an anti-inflammatory immunometabolite produced from cis-aconitate by the enzyme encoded by immune-responsive gene 1 (IRG1, also known as ACOD1) (244), modulates inflammatory signaling and cellular metabolism partly through the electrophilic modification of redox-sensitive cysteine residues (245, 246). In EAE, treatment with the cell-permeable itaconate derivative 4-octyl itaconate is reported to attenuate disease severity by suppressing pro-inflammatory microglial activation while promoting NRF2-dependent cytoprotective signaling (247). In complementary studies, electrophilic itaconate and fumarate derivatives inhibited both the priming and activation of the canonical NLRP3 inflammasome in macrophages and microglia (248).

Previous studies demonstrated that iNOS-derived NO• contributes to the regulation of intracellular itaconate metabolism during macrophage activation (249, 250). Mechanistically, NO• inhibits mitochondrial aconitase 2 (ACO2), disrupting tricarboxylic acid (TCA) cycle flux at the citrate-to-isocitrate step. Although this metabolic remodeling initially promotes citrate accumulation, sustained ACO2 inhibition is believed to limit the availability of cis-aconitate for IRG1-mediated itaconate synthesis, thereby restricting excessive itaconate accumulation (249, 250). More recently, an additional NO•-independent mechanism has been identified in which mitochondrial iNOS directly interacts with IRG1 in a conformation-dependent manner, thereby suppressing IRG1 enzymatic activity independently of NO• production (251). Collectively, these findings suggest that iNOS regulates macrophage immunometabolism through at least two mechanistically distinct pathways: a canonical NO•-dependent pathway that modulates redox-sensitive metabolic enzymes and inflammatory signaling, and a newly identified NO•-independent pathway that directly limits intracellular itaconate production through the inhibition of IRG1. Given that itaconate suppresses inflammatory activation while promoting cytoprotective and inflammation-resolving programs, the latter mechanism may indirectly influence macrophage polarization, although this possibility remains speculative at present. Because GSNO-mediated S-nitrosylation represents a major downstream signaling mechanism of NO• biology, it is conceivable that GSNO may also influence macrophage immunometabolism. However, whether the IRG1–itaconate axis contributes to macrophage polarization in MS and EAE, and whether this pathway is regulated by GSNO-mediated S-nitrosylation, remain unknown.

At present, the direct mechanisms by which GSNO-mediated S-nitrosylation regulates M2 polarization remain poorly defined. *In vitro* studies using iNOS-deficient macrophages showed markedly enhanced M1 polarization with little effect on M2 differentiation, suggesting that endogenous NO• plays a more prominent role in restraining M1 activation than in directly driving the M2 phenotype (83). In contrast, GSNO treatment reduced M2 macrophage abundance in a murine model of castration-resistant prostate cancer (252), indicating that the effects of GSNO on macrophage polarization are likely disease- and tissue-dependent. In EAE, pharmacological inhibition of GSNOR with N6022 increased CNS infiltration of Th2 and Treg cells (81), which produce IL-4, IL-13, and IL-10, cytokines known to favor M2 polarization (237–239) (**Figure 3B**). Collectively, these findings suggest that GSNO shapes macrophage polarization through both direct redox signaling and indirect cytokine-mediated effects, with their relative contributions and possible interplay with the IRG1–itaconate axis remaining to be determined in MS and EAE.

### 4.3 S-nitrosylation-mediated mechanisms for inflammatory gene expression

Inflammatory conditions lead to iNOS-derived NO• production, which exerts both cytotoxic and anti-inflammatory effects (32). Among its regulatory mechanisms, S-nitrosylation plays a pivotal role, as GSNOR inhibition elevates GSNO levels and reshapes S-nitrosylation patterns, particularly on transcription factors such as NF-κB, STAT3, HIF-1α, and Nrf2, thereby suppressing inflammatory gene expression (253). NF-κB is a master transcription factor central to the regulation of proinflammatory gene expression. It functions as a dimeric complex composed of five family members: p65 (RelA), RelB, c-Rel, p50, and p52, with the p65/p50 heterodimer being the principal form activated in response to inflammatory stimuli (254). In resting cells, p65/p50 dimers are retained in the cytoplasm in an inactive state by binding to inhibitory proteins known as inhibitors of κB (IκB) (255). Upon exposure to proinflammatory signals such as lipopolysaccharide (LPS) or TNF-α, IκB is phosphorylated by the IκB kinase (IKK) complex, comprising two catalytic subunits (IKKα and IKKβ) and a regulatory subunit (NEMO or IKKγ), leading to its ubiquitination and proteasomal degradation (**Figure 3C**). This process liberates the NF-κB dimers, allowing their translocation into the nucleus and activation of target genes (256, 257).

Among the IKK components, IKKβ is recognized as the predominant kinase responsible for activating the p65/p50 pathway in response to proinflammatory cytokines (258–260). Notably, Cys179 within the activation loop of IKKβ is essential for its kinase activity and for phosphorylation of IκB (261). NO• has been shown to inhibit IKKβ through S-nitrosylation of this cysteine residue, thereby attenuating NF-κB-mediated proinflammatory gene expression (228, 262–265). Furthermore, iNOS-derived NO• also directly inhibits NF-κB activity by S-nitrosylating Cys62 of the p50 subunit and Cys38 of the p65 subunit (266), both of which reside within the DNA-binding Rel homology domain (RHD) (266, 267) (**Figure 3C**).

The STAT family comprises transcription factors, including STAT1, STAT2, STAT3, STAT4, STAT5A, STAT5B, and STAT6, which play crucial roles in cytokine and growth factor signaling. In their inactive state, STAT proteins reside in the cytoplasm. Upon stimulation by cytokines or growth factors, specific cell surface receptors activate associated tyrosine kinases (TKs), particularly members of the Janus kinase (JAK) family: JAK1, JAK2, JAK3, and TYK2. These kinases phosphorylate a conserved tyrosine residue on STAT proteins, leading to their formation of either homodimers or heterodimers. The phosphorylated STAT dimers then translocate to the nucleus, where they bind to specific DNA sequences and regulate the transcription of target genes (268–271).

Among the STAT family, STAT1 and STAT3 are particularly important in mediating inflammatory and immune responses (272). STAT1 is predominantly activated by interferons, particularly IFN-γ through JAK1 and JAK2 and IFN-α/β through JAK1 and TYK2. Upon phosphorylation, STAT1 enters the nucleus and induces genes critical for antiviral defense and inflammation (273) (**Figure 3C**). Notably, IFN-γ-driven STAT1 activation leads to M1 macrophage polarization and the production of pro-inflammatory cytokines such as TNF-α and IL-12 (273). The precise role of NO• in the regulation of STAT1-mediated signaling remains incompletely understood. In macrophages, stimulation with lipopolysaccharide (LPS) and IFN-γ induces iNOS expression, resulting in elevated NO• production. This leads to tyrosine nitration of STAT1, which impairs phosphorylation at the critical Tyr701 residue and consequently inhibits downstream signaling (274). Additionally, other studies have reported that S-nitrosylating agents, such as SNAP and GSNO, suppress STAT1 phosphorylation and impair its binding to γ-interferon activation site (GAS) elements in macrophages and endothelial cells (243, 275) (**Figure 3C**). However, the specific sites on STAT1 where tyrosine nitration or S-nitrosylation occurs, as well as the precise effects of these modifications on STAT1 activity and STAT1-mediated inflammatory gene expression in the context of MS and EAE, remain unknown.

STAT3 activation occurs in a cytokine-specific manner via JAK family kinases, including JAK1, JAK2, and TYK2. For instance, IL-6 engages a receptor complex composed of the IL-6Rα and the signal-transducing gp130 chain, which facilitates the recruitment and activation of JAK1 and JAK2, leading to STAT3 phosphorylation at Tyr705 and its subsequent activation (276) (**Figure 3C**). Activated STAT3 dimerizes and translocates to the nucleus, where it induces the transcription of genes that contribute to the pathogenesis of chronic inflammation (277). Kim et al. (91) previously demonstrated that, under inflammatory conditions, IL-6–induced STAT3 activation in microglia is suppressed by either iNOS-derived NO• or exogenous GSNO treatment. Notably, GSNO does not affect JAK activity or the gp130 receptor; rather, it inhibits STAT3 phosphorylation through S-nitrosylation of STAT3 at Cys259 (91) (**Figure 3C**). As STAT3 is pivotal in driving the proliferation and survival of inflammatory cells by regulating genes related to cell cycle progression and anti-apoptotic signaling during immune responses (278), GSNO-mediated inhibition of STAT3 leads to suppression of IL-6-induced microglial proliferation (91). Similar findings have been reported in head and neck squamous cell carcinoma and multiple myeloma, where S-nitrosylating agents inhibit survival and proliferation of cancer cells through STAT3 suppression (279, 280). In addition to its role in inflammation and cancer, STAT3 also plays a crucial role in Th17 polarization and effector function (281). Nath et al. (80) demonstrated that GSNO treatment significantly reduced IL-17 production in MOG_35-55_- and PLP_139-151_-primed T cells by inhibiting STAT3 phosphorylation and RORγt expression, key regulators of Th17 differentiation. Consistently, in EAE models, both prophylactic and therapeutic oral administration of GSNO attenuated disease severity by suppressing IL-17-mediated neuroinflammation, underscoring the therapeutic potential of S-nitrosylation-dependent regulation of STAT3 in modulating Th17-driven autoimmunity (80).

## 5 S-nitrosylation-mediated regulation of oxidative stress and mitochondrial dysfunction in MS/EAE

MS is marked by disrupted redox homeostasis and mitochondrial dysfunction, which together contribute to inflammation, demyelination, and neurodegeneration. Activated microglia and infiltrating immune cells generate excessive levels of reactive oxygen and nitrogen species (ROS/RNS), resulting in widespread oxidative damage to DNA, proteins, and lipids, particularly within chronically demyelinated lesions (282, 283). Mitochondria are not only damaged by oxidative stress but also amplify it, ultimately leading to energy failure and neuronal loss. In MS, although endogenous antioxidant defenses are upregulated in response to oxidative stress, they are often insufficient to counteract sustained oxidative stress (282–284). Recent studies have identified S-nitrosylation as a critical redox-sensitive regulatory mechanism in pathological conditions. By attenuating ROS/RNS-induced toxicity, preserving mitochondrial integrity, and restoring redox balance, S-nitrosylation may help mitigate oxidative damage. This section discusses the emerging role of S-nitrosylation in regulating oxidative stress and mitochondrial dysfunction in MS and its potential as a therapeutic target.

### 5.1 GSNO/S-nitrosylation-mediated regulation of NO•/RNS synthesis

During inflammation, NO• generated by iNOS not only promotes tissue damage through ONOO^-^ formation but also engages in a self-limiting feedback loop by S-nitrosylating iNOS at Cys109 near the oxygenase domain. This modification disrupts the dimer–monomer equilibrium, destabilizing the active dimer, reducing catalytic efficiency, and lowering NO• output (285–287). Additionally, ONOO^-^-mediated tyrosine nitration has been reported as another negative regulatory mechanism of iNOS activity (288, 289). Unlike other NOS isoforms, iNOS expression is markedly upregulated during inflammation and is subject to limited intrinsic regulatory control. Accordingly, these autoinhibitory feedback mechanisms are thought to play an important role in moderating NO• overproduction and reducing nitrosative stress in sepsis and other inflammatory conditions.

In contrast to iNOS, eNOS and nNOS are constitutively expressed and generate low, tightly regulated NO• levels that support vascular and neuronal function. These isoforms generally serve protective or regulatory roles; however, under pathological conditions, uncoupling from essential cofactors such as L-arginine and tetrahydrobiopterin (BH_4_) can lead to O_2_•^-^ production, shifting their role from physiological regulators to drivers of oxidative and nitrosative stress (290). In this context, S-nitrosylation-mediated feedback regulation of eNOS and nNOS may be critical for mitigating such pathological outcomes.

In endothelial cells, NO• induces S-nitrosylation of eNOS at Cys94 and Cys99, two critical residues at the homodimer interface required for proper dimer formation and enzymatic activation (291, 292). Additional S-nitrosylation occurs at Cys661, Cys802, and Cys1114, which are located near the cofactor-binding domains for FMN, FAD, and NADPH; however, the precise functional roles of these sites remain incompletely understood (293). NO• can also regulate eNOS activity indirectly through S-nitrosylation of heat shock protein 90 (Hsp90), which impairs its ATPase activity and disrupts the Hsp90-eNOS complex. This disruption reduces eNOS phosphorylation at Ser1177, a modification required for full enzymatic activation (294).

Similarly, nNOS is subject to feedback inhibition via S-nitrosylation, predominantly at Cys331 and Cys336, which are located near the NADPH-binding and heme domains. Modification of these residues disrupts electron transfer and enzyme dimer stability, leading to autoinhibition of nNOS and limiting excess NO• production, thereby preventing nitrosative stress (295, 296). Collectively, available evidence indicates that S-nitrosylation of eNOS and nNOS can attenuate their NO•-producing capacity. Nonetheless, it remains unclear whether NO• or GSNO modulates O_2_•^-^ generation via S-nitrosylation under uncoupled conditions, as this has not yet been experimentally established. Further studies will be required to clarify this potential regulatory mechanism.

**Figure 4:**
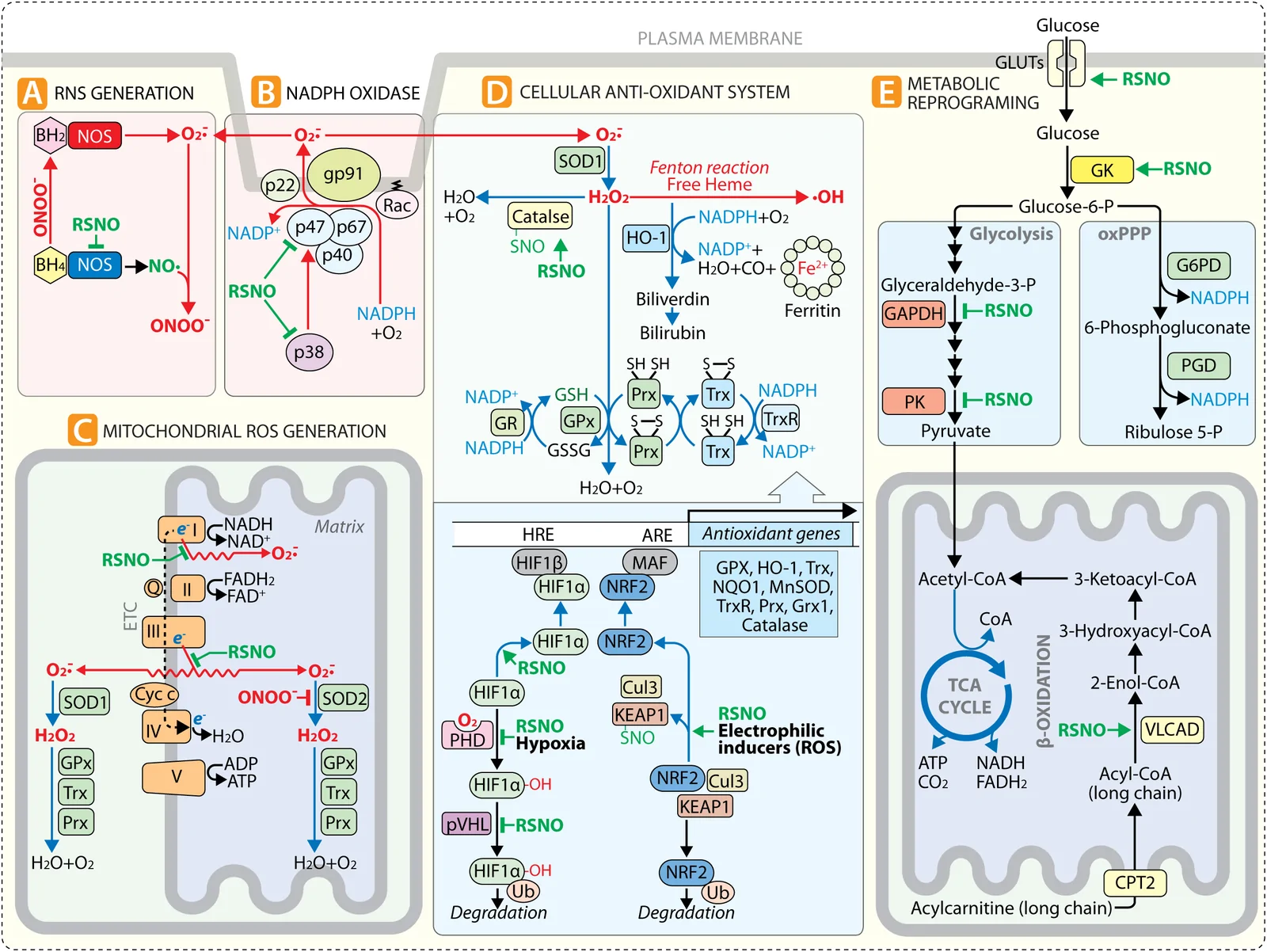
S-nitrosylation-mediated regulation of oxidative stress and mitochondrial dysfunction in MS and EAE. **(A)** Regulation of reactive nitrogen species (RNS) production by nitric oxide synthase (NOS) isoforms. In MS and EAE, inflammatory stimuli upregulate inducible NOS (iNOS), leading to excessive production of nitric oxide (NO•) and its toxic derivative peroxynitrite (ONOO^-^). S-nitrosylating agents (RSNO), including S-nitrosoglutathione (GSNO), inhibit iNOS, endothelial NOS (eNOS), and neuronal NOS (nNOS) via site-specific S-nitrosylation, thereby attenuating NO• and ONOO^-^ levels and mitigating nitrosative stress and tissue injury. **(B)** Modulation of nicotinamide adenine dinucleotide phosphate (NADPH) oxidase-derived reactive oxygen species (ROS) production. Activated phagocytes upregulate NADPH oxidase 2 (NOX2), a multi-subunit enzyme complex composed of membrane-bound and cytosolic components, including phagocyte oxidase (phox) subunits. Among these, the cytosolic subunit p47^phox^ (phagocyte oxidase 47 kDa) is critical for complex activation. S-nitrosylation of p47^phox^ disrupts NOX2 complex assembly and activity, thereby reducing superoxide (O_2_•^-^) production and limiting oxidative damage in inflamed tissues. **(C)** Mitochondrial ROS production. In demyelinated lesions of MS, mitochondrial dysfunction leads to excessive O2•^-^ generation due to electron leakage from electron transport chain (ETC) complexes I, II, and III. This disrupts oxidative phosphorylation, impairs ATP production, and exacerbates oxidative stress. RSNO attenuates mitochondrial ROS generation by S-nitrosylating ETC components, such as Cys39 on the ND3 subunit of Complex I, thereby reducing electron leakage and O_2_•^-^ output. **(D)** Cellular antioxidant defense system regulation. S-nitrosylation enhances antioxidant defense by modifying Kelch-like ECH-associated protein 1 (Keap1), disrupting its interaction with nuclear factor erythroid 2–related factor 2 (Nrf2) and preventing its proteasomal degradation. Stabilized Nrf2 translocates into the nucleus, where it dimerizes with small Maf proteins and binds to antioxidant response element (ARE) sequences in the promoters of redox-regulating genes. This induces the expression of key antioxidant enzymes, including catalase, glutathione peroxidase (GPx), peroxiredoxin (Prx), thioredoxin (Trx), and heme oxygenase-1 (HO-1), thereby enhancing the cellular capacity to buffer oxidative stress. In parallel, S-nitrosylation also stabilizes hypoxia-inducible factor 1-alpha (HIF-1α) by inhibiting its hydroxylation and proteasomal degradation. Stabilized HIF-1α promotes transcription of antioxidant and metabolic genes, including those encoding SOD2, CAT, GPx, and HO-1, further reinforcing cellular redox defense, especially under inflammatory or hypoxic conditions. **(E)** Metabolic reprogramming for the stable supply of redox cofactors (NADPH and GSH). Chronic oxidative stress in MS and EAE depletes cellular NADPH and GSH pools, impairing redox buffering. RSNOs help restore redox homeostasis by increasing glucose uptake while simultaneously suppressing glycolytic flux via S-nitrosylation of key enzymes such as glyceraldehyde-3-phosphate dehydrogenase (GAPDH) and pyruvate kinase (PK). As a result, more intracellular glucose is diverted toward the oxidative pentose phosphate pathway (oxPPP), thereby enhancing NADPH generation. In parallel, RSNOs promote fatty acid β-oxidation by S-nitrosylating very long-chain acyl-CoA dehydrogenase (VLCAD), which compensates for reduced glycolytic output by generating acetyl-CoA and sustaining adenosine triphosphate (ATP) production. Together, these metabolic adaptations maintain a stable supply of NADPH and GSH, supporting antioxidant defenses under chronic inflammatory stress.

Overall, as summarized in **Figure 4A**, NO•/GSNO-mediated S-nitrosylation inhibits NOS activity and limits excessive NO•/RNS production. Under pathological conditions involving inflammation and oxidative stress, this regulation may function as a self-limiting feedback loop that protects cells and tissues from severe RNS-mediated injury.

### 5.2 S-nitrosylation-mediated regulation of oxidative stress by NADPH oxidase

NADPH oxidase (NOX) transfers electrons from NADPH to molecular oxygen, generating large amounts of O_2_•^-^ in a process known as the oxidative burst. While this plays a critical role in eliminating invading pathogens, it can also contribute to tissue injury in pathological conditions such as atherosclerosis, hypertension, diabetes, and MS (297, 298). The NOX family comprises seven known isoforms: NOX1, NOX2, NOX3, NOX4, NOX5, and the dual oxidases DUOX1 and DUOX2. Among them, NOX2, also known as phagocyte oxidase (phox), is the most extensively studied isoform and is considered the most pathologically significant, particularly due to its role in immune-mediated oxidative stress.

NOX2 activation requires the assembly of a multi-subunit enzyme complex. Its membrane-bound core consists of gp91^phox^ (the catalytic subunit, also known as cytochrome b_245_ β chain, encoded by CYBB) and p22^phox^, which together form flavocytochrome b_558_. Upon stimulation by pathogens or cytokines, the key regulatory subunit p47^phox^ orchestrates the translocation of cytosolic components, including p67^phox^ and p40^phox^, to the membrane, thereby initiating assembly of the active NOX2 complex. Full activation also requires the small GTPase Rac (predominantly Rac2 in phagocytes and Rac1 in non-phagocytic cells) (299) (**Figure 4B**).

NO• is known to inhibit NOX2-derived O_2_•^-^ production by S-nitrosylating the regulatory subunit p47^phox^ at Cys196. This modification alters its conformation and enhances its phosphorylation by casein kinase 2 (CK2), ultimately impairing p47phox’s ability to translocate to the membrane and assemble the functional NOX2 complex (300, 301) (**Figure 4B**). Additionally, S-nitrosylation of p38 MAPK reduces its kinase activity, leading to decreased p47^phox^ activity. This indirectly impairs NOX2 complex assembly, adding another layer of redox-based control over O_2_•^-^ production (297, 302).

### 5.3 S-nitrosylation-mediated regulation of oxidative stress in mitochondria

In MS, ROS from inflammatory cells damage mitochondrial DNA (mtDNA), electron transport chain (ETC) proteins, and lipids (303–305). Dysfunctional mitochondria then generate additional ROS, amplifying oxidative stress in a vicious cycle (306, 307). This further depletes ATP, particularly in demyelinated axons that have high energy demands (308, 309), resulting in disturbed ion homeostasis, calcium accumulation, and activation of cell death pathways (310, 311). Oxidative stress also hinders oligodendrocyte maturation and remyelination by suppressing key mitochondrial transcripts (312, 313). Postmortem MS brains show reduced ETC activity, mtDNA deletions, and elevated markers of mitochondrial stress (308, 310, 314, 315). Together, these findings highlight mitochondrial dysfunction as both a driver and consequence of neurodegeneration in MS.

Mechanistically, O_2_•^-^ is generated in mitochondria through premature electron leakage from ETC complexes I (NADH dehydrogenase), II (succinate dehydrogenase), and III (ubiquinol-cytochrome c reductase), resulting in incomplete one-electron reduction of O_2_ (316). Under normal conditions, low levels of O_2_•^-^ are efficiently dismuted to hydrogen peroxide (H_2_O_2_) by manganese superoxide dismutase (MnSOD or SOD2) in the matrix and copper/zinc superoxide dismutase (CuZnSOD or SOD1) in the intermembrane space. However, under pathological conditions, O_2_•^-^ production can exceed the neutralizing capacity of endogenous SODs, thus leading to the accumulation of ROS, mitochondrial damage, and the initiation of downstream cell death pathways (317, 318) (**Figure 4C**).

NO• influences mitochondrial function through both physiological and pathological mechanisms. Under normal conditions, NO• is known to modulate respiration by reversible inhibition of ETC complexes I, III, and IV (cytochrome c oxidase, COX) (319–323). For example, NO• binds to the heme moiety at the catalytic center of complex IV, temporarily reducing its activity and thereby decreasing electron flux, oxygen consumption, and ATP synthesis (324–326). Under pathological conditions, however, when excessive mitochondrial O_2_•^-^ production coincides with high NO• levels, the two radicals rapidly react to form ONOO^-^, at a rate greater than that of O_2_•^-^ dismutation to H_2_O_2_ (305). ONOO^-^ induces further mitochondrial dysfunction by nitrating key proteins (e.g., NADH dehydrogenase and succinate dehydrogenase), oxidizing essential lipids (e.g., cardiolipin), and damaging mitochondrial DNA, ultimately exacerbating bioenergetic failure and promoting cell death (327).

In mitochondria, aberrant S-nitrosylation can disrupt protein folding and promote mitochondrial fragmentation, ultimately contributing to energy failure and cell death (328). However, emerging evidence also underscores the protective role of physiological S-nitrosylation, particularly in maintaining mitochondrial homeostasis by modulating ROS production and preserving redox balance. For example, physiological micromolar concentrations of GSNO markedly suppress mitochondrial O_2_•^-^ and H_2_O_2_ production arising from electron leakage at complexes I and III during succinate- or NAD-linked substrate-driven ETC activity (329, 330). Similarly, S-nitroso-N-acetylpenicillamine (SNAP), a synthetic S-nitrosylating agent, has been shown to inhibit electron transfer at complex I through S-nitrosylation of Cys39 on the ND3 subunit of NADH dehydrogenase, resulting in reduced ROS production and enhanced protection against ischemic injury (331). Moreover, S-nitrosylation exerts a protective effect by suppressing the peroxidase activity of the cytochrome c-cardiolipin complex, thereby preventing cardiolipin oxidation under oxidative stress, an early event in mitochondria-mediated apoptosis (332).

Collectively, these findings suggest that S-nitrosylation at physiological concentrations is essential for preserving mitochondrial function and mitigating oxidative stress (**Figure 4C**). However, under pathological conditions, excessive levels of GSNO (in the millimolar range) or other S-nitrosylating agents have been reported to exert deleterious effects, including persistent complex I modification that enhances O_2_•^-^ production (333), as well as Ca^2+^-induced opening of the mitochondrial permeability transition pore (MPTP) via modification of critical mitochondrial proteins such as adenine nucleotide translocase (ANT), voltage-dependent anion channel (VDAC), and ATP synthase (334). As S-nitrosylation confers protective effects at physiological levels but becomes detrimental when excessive, its precise regulation is crucial for maintaining mitochondrial integrity and function.

### 5.4 S-nitrosylation-mediated regulation of the cellular antioxidant system

O_2_•^-^ is a key initiator of oxidative stress. While moderately reactive under physiological conditions, excess O_2_•^-^ can disrupt mitochondrial Fe-S clusters (e.g., aconitase, succinate dehydrogenase), causing enzyme inactivation, ferrous iron (Fe^2+^) release, and mitochondrial dysfunction (317, 318). Normally, O_2_•^-^ is converted to H_2_O_2_ by SOD1 in the cytoplasm and intermembrane space and SOD2 in the mitochondrial matrix (335) (**Figure 4D**). Although less reactive than O_2_•^-^, H_2_O_2_ accumulation, together with increased release of redox-active Fe^2+^ from heme degradation under pathological conditions, can drive Fenton-type reactions, generating highly reactive hydroxyl radicals (HO•) (336). To limit this threat, cells activate antioxidant defenses that sequester free heme and Fe^2+^ while degrading excess H_2_O_2_.

Under stress, free heme itself acts as a potent pro-oxidant but is degraded by heme oxygenase-1 (HO-1) into biliverdin, carbon monoxide (CO), and Fe^2+^ (337). The released Fe^2+^ is rapidly sequestered by ferritin, thereby preventing iron-driven Fenton chemistry and oxidative injury, while biliverdin is subsequently reduced to bilirubin; both bile pigments provide potent antioxidant protection (338, 339) (**Figure 4D**). H_2_O_2_ is further detoxified by catalase in peroxisomes, as well as by glutathione peroxidases (GPx1-GPx7) and peroxiredoxins (Prx1-Prx6) distributed across multiple cellular compartments, including the cytoplasm. GPxs use GSH to convert H_2_O_2_ into water and detoxify lipid hydroperoxides by converting them into alcohols. In this process, GSH is oxidized to glutathione disulfide (GSSG), which is then converted back to GSH by glutathione reductase (GR) in a reaction that requires NADPH (340, 341) (**Figure 4D**). Prxs catalyze similar reductions with thioredoxin (Trx) as the electron donor, which is regenerated by thioredoxin reductase (TrxR) in an NADPH-dependent process (342, 343) (**Figure 4D**).

In MS and EAE, antioxidant enzymes such as SOD, catalase, GPx, and GR are upregulated; however, oxidative damage in the CNS persists due to limited enzyme expression, rapid GSH depletion by reactive aldehydes like acrolein, which also inhibits GR, and sustained ROS/RNS production from infiltrating immune cells (344, 345). NO• has long been recognized not only as a key player in ROS/RNS chemistry but also as a crucial modulator of endogenous antioxidant defenses. For instance, NO• influences antioxidant enzymes through post-translational modifications. One notable example is the catalase, whose activity is modulated through S-nitrosylation. Silencing of GSNOR, which elevates intracellular levels of GSNO, enhances the S-nitrosylation of catalase at Cys401, Cys642, and Cys653, of which Cys401 plays a particularly critical role in regulating its enzymatic activity. This post-translational modification markedly enhances catalase function, thereby contributing to a reduction in oxidative stress (346) (**Figure 4D**).

Beyond post-translational regulation, NO• also modulates redox homeostasis at the transcriptional level by targeting key transcription factors such as nuclear factor erythroid 2-related factor 2 (Nrf2) and hypoxia-inducible factor-1α (HIF-1α). Through S-nitrosylation, NO• can stabilize and enhance the transcriptional activity of these regulators, thereby promoting the expression of diverse antioxidant and cytoprotective genes (**Figure 4D**) (347).

Under basal conditions, Nrf2 is sequestered in the cytoplasm by Kelch-like ECH-associated protein 1 (Keap1) and Cullin-3, which together form an E3 ubiquitin ligase complex that targets Nrf2 for proteasomal degradation (348). Upon oxidative or electrophilic stress, specific cysteine residues on Keap1 are modified, disrupting the Keap1-Cul3 complex and allowing Nrf2 to accumulate, translocate to the nucleus, and dimerize with small Maf proteins (349, 350). This leads to the activation of antioxidant response element (ARE)-driven genes, including HO-1, NQO1, SOD2, catalase, Trx1, Grx1, GPx4, and GSTs (351–356). Importantly, NO• can potentiate this pathway by S-nitrosylating Keap1, thereby preventing its repressive interaction with Nrf2 and enhancing antioxidant gene transcription (353, 354, 357) (**Figure 4D**).

HIF-1α is primarily responsible for cellular adaptation to hypoxia but also contributes to redox regulation by inducing antioxidant enzymes such as SOD2, CAT, GPx4, Prx1, Prx5, and HO-1 (347, 358), as well as by promoting glutathione biosynthesis (359). Under normoxia, HIF-1α is hydroxylated by prolyl hydroxylase domain protein 2 (PHD2), which targets it for ubiquitination and proteasomal degradation (167–169). NO• can stabilize HIF-1α even under normoxic conditions by S-nitrosylating both HIF-1α and PHD2, thereby preventing hydroxylation-dependent degradation and enabling sustained transcription of HIF-1α target genes (173–175) (**Figure 4D**).

Among the antioxidant genes regulated by Nrf2 and HIF-1α, GPx4 is particularly important because it uses GSH to detoxify lipid hydroperoxides and prevent membrane phospholipid peroxidation (360), thereby protecting cells against ferroptosis, an iron-dependent form of regulated cell death driven by excessive lipid peroxidation In MS and EAE, ferroptosis contributes to oligodendrocyte loss, neuronal injury, and disease progression through GSH depletion, reduced GPx4 expression and activity, iron accumulation, and lipid peroxidation (361, 362). Accordingly, inhibiting ferroptosis or restoring GPx4 activity attenuates neuroinflammation and demyelination in experimental models, supporting ferroptosis as a potential therapeutic target in MS (361, 363).

The relationship between NO• and ferroptosis appears to be highly context-dependent. On the one hand, excessive NO• readily reacts with O_2_•^-^ to generate ONOO^-^, a potent oxidant that promotes lipid peroxidation and may contribute to ferroptotic cell death under conditions of sustained nitrosative stress (364). On the other hand, emerging evidence suggests that GSNO-mediated S-nitrosylation may indirectly counteract ferroptosis through multiple mechanisms. As discussed above, S-nitrosylation of Keap1 activates the Nrf2 antioxidant pathway (353, 354, 357), whereas stabilization of HIF-1α further supports cellular antioxidant capacity by inducing antioxidant enzymes and glutathione biosynthesis (173–175). In addition, S-nitrosylation has been also proposed to modulate ferroptosis-associated signaling pathways, including the ASK1-P38/JNK MAPK cascade, although current evidence remains largely indirect and is derived primarily from cancer models (365). Consistent with an overall protective role of NO•, NO• donors suppress ferroptosis by terminating lipid peroxidation chain reactions independently of the canonical GPx4 pathway (366). Moreover, iNOS-derived NO• confers resistance to ferroptosis even under conditions of GPx4 deficiency (367). Whether GSNO-mediated S-nitrosylation directly regulates the core ferroptotic machinery in MS and EAE remains unknown and thus warrants further investigation.

In summary, S-nitrosylation is a critical mechanism by which NO• regulates cellular antioxidant defenses at both the enzymatic and transcriptional level. These mechanisms may also limit ferroptotic cell death by reducing oxidative stress, enhancing glutathione-dependent antioxidant capacity, and inducing cytoprotective gene expression, though whether GSNO-mediated S-nitrosylation directly regulates core ferroptotic machinery remains unknown. While antioxidants have shown promise in inflammatory and oxidative disease settings, upregulating antioxidant enzymes in peripheral compartments does not necessarily protect vulnerable CNS regions such as white matter and cortex in MS, and many antioxidant compounds show limited CNS bioavailability, underscoring the need for CNS-targeted strategies (368). Recent findings in mice suggest that systemic administration of the GSNOR inhibitor N91115 can elevate S-nitrosothiol levels within the CNS, potentially enhancing endogenous antioxidant responses in MS or EAE (53). However, these observations require further experimental validation to confirm their therapeutic relevance and efficacy.

### 5.5 S-nitrosylation-mediated metabolic reprogramming to coordinate energy production and redox balance

NADPH plays a central role in maintaining cellular redox homeostasis by providing reducing equivalents to GR and TrxR, which regenerate key antioxidants such as GSH and Trx. Simultaneously, it fuels NOX and NOS, thereby supporting the production of ROS and RNS. This dual functionality enables NADPH to mediate a fine balance between the generation of oxidative/nitrosative stress and antioxidant defense, allowing cells to dynamically regulate their redox state in response to fluctuating conditions (369).

In MS and EAE, redox imbalance is particularly evident in brain white matter, where reduced GSH levels have been detected in patients, reflecting impaired antioxidant defenses (370, 371). Under these pathological conditions, a stable supply of NADPH is essential to counteract oxidative and nitrosative stress. NADPH is mainly produced through the oxidative pentose phosphate pathway (oxPPP), which competes with glycolysis for glucose as a common substrate (**Figure 4E**). Thus, increased demand for NADPH creates a metabolic trade-off: greater oxPPP flux may reduce glycolytic ATP production, and vice versa. As a result, glucose metabolism is reprogrammed to prioritize either energy generation or redox balance depending on cellular demands, a shift that becomes especially relevant in the inflammatory and degenerative environment of MS.

NO• is a key modulator of glucose metabolism. It promotes the expression and membrane localization of glucose transporters (GLUT1 and GLUT4) (372–374), thereby facilitating increased glucose uptake (372, 375, 376) (**Figure 4E**). Intriguingly, NO• exhibits a dose-dependent dual effect on glycolysis: at low concentrations, it enhances glycolysis, partly through activation of 5′-AMP-activated protein kinase (AMPK) by increasing AMP levels or promoting phosphorylation of AMPK-α1 at Thr172 (377, 378). NO• also stabilizes HIF-1α, which enhances glycolysis by upregulating glucose transporters (GLUT1, GLUT3) and key glycolytic enzymes, including hexokinase 2 (HK2), phosphofructokinase (PFK), and glyceraldehyde-3-phosphate dehydrogenase (GAPDH), thereby accelerating glucose metabolism through the glycolytic pathway (379).

However, in inflammatory states, excessive NO• produced by iNOS negatively impacts glycolytic flux. It inhibits key enzymes like GAPDH through S-nitrosylation at Cys149, which promotes its nuclear translocation and disrupts cytoplasmic glycolytic metabolism (380–384). Similarly, pyruvate kinase M2 (PKM2), essential for the final step in glycolysis in embryonic cells, activated macrophages, and CD4^+^ T cells, is inhibited by S-nitrosylation, which prevents the formation of its active tetramer (385, 386) (**Figure 4E**). These findings suggest that, under pathological conditions, excessive NO• redirects glucose flux from glycolysis to the oxPPP, thereby increasing NADPH production and antioxidant capacity to buffer nitrosative stress (385, 387, 388).

Importantly, while glucose is indispensable for fueling the oxPPP for NADPH synthesis, ATP production can be supported by alternative fuels like ketone bodies and amino acids under conditions of glucose scarcity (389). Interestingly, NO• has also been shown to promote energy production through lipid metabolism. Specifically, it enhances the activity of very long-chain acyl-CoA dehydrogenase (VLCAD), a rate-limiting enzyme in mitochondrial fatty acid β-oxidation, via S-nitrosylation at Cys238, thus supporting acetyl-CoA and ATP production through this parallel route (390) (**Figure 4E**).

Metabolic reprogramming is now recognized as a major determinant of immune-cell differentiation and function. Accordingly, NO•-dependent metabolic changes may also influence adaptive immune responses relevant to MS pathogenesis. In MS, the balance between Th17 cells and Treg cells, which have opposing roles in immunity and inflammation, is crucial for immune homeostasis (391). Notably, Th17 cells rely mainly on aerobic glycolysis, whereas Treg cells depend on fatty acid oxidation and oxidative phosphorylation to meet their metabolic demands (392–394). Recent findings show that GSNO or reversible inhibition of GSNOR (e.g., with N6022 and N91115) suppresses Th17 differentiation and effector functions while enhancing those of Treg cells in EAE mice (53, 80, 81, 134, 163). These distinct metabolic requirements raise the possibility that GSNO-mediated signaling contributes to immune regulation by altering cellular metabolic programs. However, whether GSNO regulates Th17 and Treg cell polarization through metabolic reprogramming, potentially by modulating glycolysis and fatty acid β-oxidation, remains unclear and warrants further investigation.

Because NADPH-dependent redox pathways operate in virtually all metabolically active tissues, the metabolic effects of GSNOR inhibition are unlikely to be restricted to CNS-infiltrating immune cells. Systemic GSNOR inhibition may therefore influence redox homeostasis in peripheral tissues with high metabolic demand, including the liver, skeletal muscle, and erythrocytes, where NADPH-dependent antioxidant systems are essential for maintaining cellular function. Such systemic effects may influence not only cellular antioxidant defenses but also tissue-specific metabolic functions, highlighting the importance of evaluating potential off-target physiological consequences during chronic GSNOR inhibition. Consistent with this possibility, preclinical toxicology studies of the GSNOR inhibitor N6022 reported mild, redox-related hepatotoxicity at high doses, whereas lower doses were well tolerated (395). Although these findings suggest the existence of a therapeutic window, the systemic metabolic consequences of chronic GSNOR inhibition have not been systematically evaluated and remain an important consideration for long-term safety assessment.

In summary, metabolic reprogramming is fundamental to coordinating cellular energy production and redox homeostasis, with NADPH serving as a central hub linking oxidative stress to antioxidant defense. Through modulation of glycolysis, the oxidative pentose phosphate pathway, and fatty acid oxidation, GSNO-mediated S-nitrosylation may shape immune cell metabolism and function while maintaining redox balance. At the same time, because these metabolic pathways are broadly utilized across multiple organs, future studies should define the systemic metabolic consequences, therapeutic window, and tissue-specific safety profile of chronic GSNOR inhibition to maximize immunomodulatory efficacy while preserving physiological redox homeostasis.

## 6 Summary, conclusions, and future perspectives

MS is a chronic autoimmune disorder of the CNS that requires lifelong management. Although DMTs have substantially improved disease management, each therapeutic class possesses inherent limitations. **Table 1** provides an overview of the mechanisms of action, major advantages, and limitations of currently available and emerging DMTs for MS. Biologic therapies, including anti-CD20 monoclonal antibodies, natalizumab, interferon-β, and glatiramer acetate, provide targeted immunomodulation and substantial clinical efficacy but generally require parenteral administration and may be associated with prolonged immune cell depletion, immunogenicity, or infusion-related complications (396–407). More recently, cytokine-targeted biologics, such as anti-IL-17/IL-23 therapies, have demonstrated remarkable efficacy in psoriasis and related autoimmune diseases; however, their clinical benefit in MS has remained limited, suggesting that selective blockade of the IL-17/Th17 pathway alone may be insufficient to control disease progression (72, 408). In contrast, orally available small-molecule therapies, including S1P receptor modulators, fumarates, teriflunomide, and cladribine, offer greater convenience but frequently rely on broad immunosuppressive mechanisms that can cause lymphopenia, increased infection risk, and other systemic adverse effects (409–418). Emerging BTK inhibitors represent another promising class of oral therapeutics by targeting both adaptive and innate immune responses, although their long-term efficacy and safety remain under investigation (419–421).

**Figure 5:**
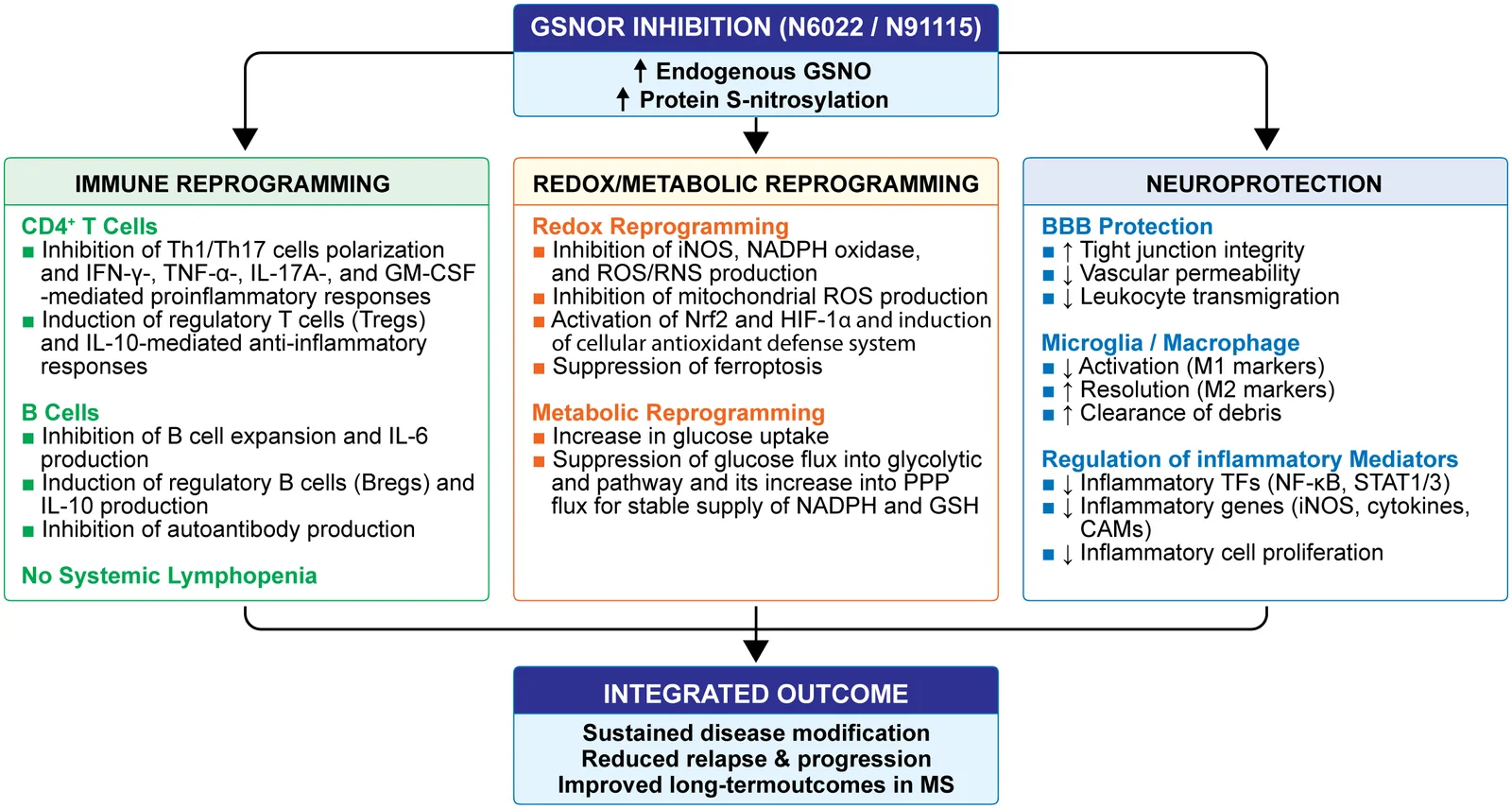
Proposed integrated model of GSNO-mediated immune reprogramming, redox/metabolic regulation, and neuroprotection in multiple sclerosis. Pharmacological inhibition of GSNOR increases endogenous GSNO levels, thereby enhancing protein S-nitrosylation and modulating multiple redox-sensitive signaling pathways. Collectively, evidence from independent mechanistic and preclinical studies suggests that GSNO and GSNOR inhibition may suppress pathogenic immune responses, oxidative stress, and neuroinflammation while promoting regulatory immune responses, antioxidant defenses, metabolic homeostasis, and neuroprotection without inducing systemic lymphopenia. This schematic summarizes the principal mechanisms discussed throughout this Review and represents a conceptual model derived primarily from EAE models. Although reversible GSNOR inhibitors have demonstrated favorable safety and tolerability in Phase I/II studies for asthma and cystic fibrosis, their therapeutic efficacy in multiple sclerosis remains to be established in future clinical trials.

Unlike these therapeutic approaches, GSNOR inhibitors represent a mechanistically distinct class of orally active small molecules that selectively enhance endogenous GSNO-mediated S-nitrosylation. By elevating intracellular GSNO, they may restore physiological NO• signaling, thereby regulating immune responses, reducing oxidative stress, and supporting mitochondrial function. Unlike many currently available biologic and oral small-molecule DMTs, which primarily rely on broad immunosuppressive or immune-depleting mechanisms, preclinical studies indicate that GSNOR inhibition reprograms immune responses by suppressing pathogenic T cells (Th1/Th17) as well as inflammatory B cells while promoting regulatory T- and B-cell populations, without inducing systemic lymphopenia (53, 80, 81, 133, 134, 163, 219, 228, 422) (**Table 1**). In addition to its immunomodulatory effects, GSNOR inhibition may also confer neuroprotective benefits through complementary mechanisms, including limiting microglial and macrophage activation, preserving blood-brain barrier integrity, upregulating antioxidant enzymes, and activating transcriptional programs such as Nrf2 and HIF-1α. Together, these multimodal actions integrate selective immune modulation, redox/metabolic regulation, and neuroprotective mechanisms, as illustrated in **Figure 5**, potentially extending the therapeutic scope beyond the primary immunomodulatory actions of most currently available DMTs.

Nevertheless, the biological effects of GSNO-mediated S-nitrosylation are highly concentration- and context-dependent. Physiological, low-micromolar concentrations of GSNO generally support antioxidant, cytoprotective, and immunomodulatory signaling, whereas excessive or sustained elevations in GSNO may lead to maladaptive S-nitrosylation of non-target proteins, impaired protein folding, mitochondrial dysfunction, and disruption of normal redox signaling networks (328, 333, 334). Because S-nitrosylation regulates numerous proteins involved in immune responses, mitochondrial respiration, apoptosis, and vascular homeostasis, prolonged or indiscriminate GSNOR inhibition may perturb systemic redox homeostasis beyond the intended therapeutic pathways. Consequently, the therapeutic benefit of GSNOR inhibition is likely to depend on achieving a balanced increase in GSNO rather than indiscriminate enhancement of global S-nitrosylation. These considerations highlight an important challenge for future clinical development. Although currently available GSNOR inhibitors have demonstrated favorable safety and tolerability profiles in preclinical studies and Phase I/II clinical trials for asthma and cystic fibrosis, the therapeutic window that optimally balances efficacy with preservation of physiological S-nitrosylation homeostasis remains to be defined. Future studies should therefore determine the optimal degree, duration, and tissue specificity of GSNOR inhibition, particularly in the context of chronic neuroinflammatory diseases such as MS.

Importantly, GSNOR inhibitors such as N91115 and N6022 are small molecules with favorable pharmacological and practical profiles. They are suitable for oral or parenteral administration, depending on the compound, avoid the logistical burdens associated with monoclonal antibody therapies, and demonstrate predictable pharmacokinetics, scalable production, and lower manufacturing costs. Both compounds have completed Phase I and II clinical trials in non-neurological diseases with favorable safety and tolerability, supporting the clinical feasibility of GSNOR inhibition in humans. However, evidence supporting the therapeutic efficacy of GSNOR inhibitors in MS remains limited to mechanistic studies, *in vitro* experiments, and EAE models, and no clinical trials have yet evaluated GSNO/GSNOR-targeted therapies in patients with MS. Therefore, well-designed clinical trials are required to determine whether the promising immunomodulatory and neuroprotective effects observed in preclinical studies can be translated into meaningful clinical benefit in patients with MS.

Taken together, current mechanistic and preclinical evidence supports the GSNO/GSNOR axis as a promising therapeutic target for MS by integrating selective immune modulation with neuroprotection. Although these conclusions are based predominantly on preclinical evidence, the favorable pharmacological properties and clinical safety profiles of GSNOR inhibitors support continued clinical development. If future clinical studies confirm these promising preclinical findings, GSNOR inhibition may represent a next-generation therapeutic strategy capable of providing durable immune reprogramming while minimizing the systemic immunosuppression associated with many current DMTs.
